# Supplementary material for: Topographic design in wearable MXene sensors with in-sensor machine learning for full-body avatar reconstruction
Source: Nat Commun. 2022 Sep 9;13:5311. doi: 10.1038/s41467-022-33021-5 (PMC9461448; doi:10.1038/s41467-022-33021-5)
Supplement: Supplementary file 1 — Supplementary Information [file 41467_2022_33021_MOESM1_ESM.pdf]

Supplementary Information

Topographic Design in Wearable MXene Sensors with In-Sensor Machine Learning for Full-Body Avatar Reconstruction

Haitao Yang[1,‡], Jiali Li[1,‡], Xiao Xiao[2,‡], Jiahao Wang[3,‡], Yufei Li[1], Kerui Li[1], Zhipeng Li[3], Haochen Yang[4], Qian Wang[1], Jie Yang[1], John S. Ho[3], Po-Len Yeh[5], Koen Mouthaan[3], Xiaonan Wang[6], Sahil Shah*[7], Po-Yen Chen*[4,8]

[1] Department of Chemical and Biomolecular Engineering, National University of Singapore, 4 Engineering Drive 4, Singapore 117585, Singapore.

[2] Department of Electrical and Electronic Engineering, Southern University of Science and Technology, Shenzhen, China.

[3] Department of Electrical and Computer Engineering, National University of Singapore, Singapore 117583, Singapore.

[4] Department of Chemical and Biomolecular Engineering, University of Maryland, College Park, MD, 20740, United States.

[5] Realtek, Singapore 609930.

[6] Department of Chemical Engineering, Tsinghua University, Beijing, 100084, China.

[7] Department of Electrical and Computer Engineering, University of Maryland, College Park, MD, 20740, United States.

[8] Maryland Robotics Center, College Park, MD, 20740, United States.

[‡] These authors contributed equally to this work.

*Email: sshah389@umd.edu (S. Shah); checp@umd.edu (P.-Y. Chen)

This PDF file includes:

Supplementary Fig. 1 to **Fig. 56**

Supplementary Note 1 to **11**

Supplementary Table 1 and **Table 7**

**Supplementary Fig. 1**	Characterization of $Ti_3C_2T_x$ MXene nanosheets and SWNTs.	S5
**Supplementary Fig. 2**	Characterizations of ps-MXene nanolayers.	S6
**Supplementary Fig. 3**	Machine for roll-to-roll production of uniaxial shrink films.	S7
**Supplementary Fig. 4**	Thermal contraction of a uniaxial PS shrink film.	S8
**Supplementary Fig. 5**	Dimension change of uniaxial shrink films after thermal contraction.	S9
**Supplementary Fig. 6**	Uniaxial shrinkage of a ps-MXene nanolayer.	S10
**Supplementary Fig. 7**	SEM image and high-resolution SEM image of a M_w nanolayer.	S11
**Supplementary Fig. 8**	High-resolution SEM images of the transition zone of a $M_{p\text{-}w\text{-}p}$ nanolayer.	S12
**Supplementary Fig. 9**	Electrical resistances of all kinds of M_n nanolayers.	S13
**Supplementary Fig. 10**	Fabrication of freestanding M_n nanolayer.	S14
**Supplementary Fig. 11**	SEM image of an as-transferred M_w nanolayer.	S15
**Supplementary Fig. 12**	AFM characterizations of M_w nanolayers.	S16
**Supplementary Fig. 13**	Schematic illustration of transferring a freestanding ps-MXene nanolayer onto a VHB^{TM} substrate.	S17
**Supplementary Fig. 14**	Fabrication of M_n sensors.	S18
**Supplementary Fig. 15**	SEM images of a M_p nanolayer under uniaxial strains.	S19
**Supplementary Fig. 16**	SEM images of a M_w nanolayer under uniaxial strains.	S20
**Supplementary Fig. 17**	SEM images of a $M_{p\text{-}w\text{-}p}$ nanolayer under uniaxial strains.	S21
**Supplementary Fig. 18**	SEM images of a $M_{w\text{-}p\text{-}w}$ nanolayer under uniaxial strains.	S22
**Supplementary Fig. 19**	Strain sensing curves of $M_{w\text{-}p\text{-}w}$ sensors with areal percentages of wrinkle-like region(s) from 5% to 75%.	S23
**Supplementary Fig. 20**	FEA simulation of four M_n nanolayers under 120% stretching.	S24
**Supplementary Fig. 21**	Cycling test of a M_p sensor under 5% strain for 20,000 cycles.	S25
**Supplementary Fig. 22**	Cycling test of a $M_{p\text{-}w\text{-}p}$ sensor under 15% strain for 20,000 cycles.	S26
**Supplementary Fig. 23**	Cycling test of a M_w sensor under 25% strain for 20,000 cycles.	S27
**Supplementary Fig. 24**	Cycling test of a $M_{w\text{-}p\text{-}w}$ sensor under 40% strain for 20,000 cycles.	S28
**Supplementary Fig. 25**	Response times of a M_p sensor in the stretching and relaxation processes.	S29

**Supplementary Fig. 26**	Stress–strain curves of all M_n sensors.	S30
**Supplementary Fig. 27**	Hysteresis curves of M_n sensors.	S31
**Supplementary Fig. 28**	Signal outputs, S_ε , of M_n sensors with varying nanolayer compositions and nanolayer thicknesses under uniaxial strains.	S32
**Supplementary Fig. 29**	Nanolayer composition, thickness, and topography effects on the ε_{max} of M_n sensors.	S33
**Supplementary Fig. 30**	Signal outputs, S_ε, of (a) M_w sensor attached on the left knee, and (b) $M_{p\text{-}w\text{-}p}$ sensor attached on the left knee, during repeated squatting movements.	S34
**Supplementary Fig. 31**	Circuit design of a wireless sensor module.	S35
**Supplementary Fig. 32**	Wireless transmission errors of wireless sensor modules.	S36
**Supplementary Fig. 33**	Voltage signals of wireless sensor module from seven M_n sensors during full-body motion monitoring.	S37
**Supplementary Fig. 34**	Circuit design of an edge sensor module.	S38
**Supplementary Fig. 35**	Comparison between 15 joint locations (from P0 to P14) determined by an edge sensor module and extracted from recorded video.	S39
**Supplementary Fig. 36**	Signal timelines of a M_p sensor on the back waist and the location trajectories of P8 joint that were extracted from the recorded video or determined from the CNN model.	S40
**Supplementary Fig. 37**	Fabrication of wrinkle-like microtextures by using pre-stretched VHB^{TM} substrates.	S41
**Supplementary Fig. 38**	Fabrication of wrinkle-like microtextures by using biaxial shrink films.	S42
**Supplementary Fig. 39**	FEA models of M_n microstructures.	S43
**Supplementary Fig. 40**	Cartesian coordinates and boundary conditions of FEA simulation.	S44
**Supplementary Fig. 41**	M_n sensors' performance under different stretching directions.	S45
**Supplementary Fig. 42**	M_w sensor performance under different stretching directions.	S46
**Supplementary Fig. 43**	$M_{p\text{-}w\text{-}p}$ sensor performance under different stretching directions.	S47
**Supplementary Fig. 44**	$M_{w\text{-}p\text{-}w}$ sensor performance under different stretching directions.	S48
**Supplementary Fig. 45**	$M_{w\text{-}p\text{-}w}$ sensor performance under different stretching directions.	S49
**Supplementary Fig. 46**	Performance comparison between M_p and M_w sensors.	S50

**Supplementary Fig. 47**	Performance comparison between $M_{p\text{-}w\text{-}p}$ and $M_{w\text{-}p\text{-}w}$ sensors.	S51
**Supplementary Fig. 48**	By increasing the nanolayer thickness from 400 to 800 nm, the wrinkle wavelength of M_w nanolayer increased from ca. 7 to 13 μm.	S52
**Supplementary Fig. 49**	Crack propagation of M_w nanolayers with varying thicknesses under various strains.	S53
**Supplementary Fig. 50**	Equivalent circuit of the connections among a M_n sensor, ADC, and standard resistor.	S54
**Supplementary Fig. 51**	Working mechanism of ANN model.	S55
**Supplementary Fig. 52**	Working mechanism of OPEN POSE program.	S56
**Supplementary Fig. 53**	Working mechanism of CNN model.	S57
**Supplementary Fig. 54**	Implementation of FIFO register for real-time computation using a CNN model.	S58
**Supplementary Fig. 55**	Increasing comfort level of wearing sensor modules.	S59
**Supplementary Fig. 56**	Reconstruction of a 3D avatar *via* MotioNet method.	S60
**Supplementary Note 1**	The effects of stretching directions on the crack propagation behaviors of M_n sensors.	S61-S63
**Supplementary Note 2**	Comparison of M_n sensors' performance.	S63-S64
**Supplementary Note 3**	The effects of nanolayer thicknesses on the M_n sensors' morphologies and crack propagation behaviors.	S64-S65
**Supplementary Note 4**	Discussion of the transmission error of wireless sensor module.	S66-S67
**Supplementary Note 5**	Artificial Neural Network (ANN) model.	S67-S68
**Supplementary Note 6**	t-distributed Stochastic Neighbor Embedding (t-SNE).	S68-S69
**Supplementary Note 7**	OPEN POSE program.	S69
**Supplementary Note 8**	Convolutional Neural Network (CNN) model.	S69-S70
**Supplementary Note 9**	Discussion of the phenomenon that sometimes the avatar animation moved ahead of the full-body motion.	S70
**Supplementary Note 10**	Improvement of the comfort levels of wearing the M_n sensor modules.	S71
**Supplementary Note 11**	Construction of 3D avatar motion.	S71
**Supplementary Table 1**	Fabrication parameters of 12 M_n sensors in Fig. 3i.	S72
**Supplementary Table 7**	Comparison of ADC-measured voltages using different standard resistor values of 5 kΩ and 100 kΩ.	S73
**Supplementary References**		S74

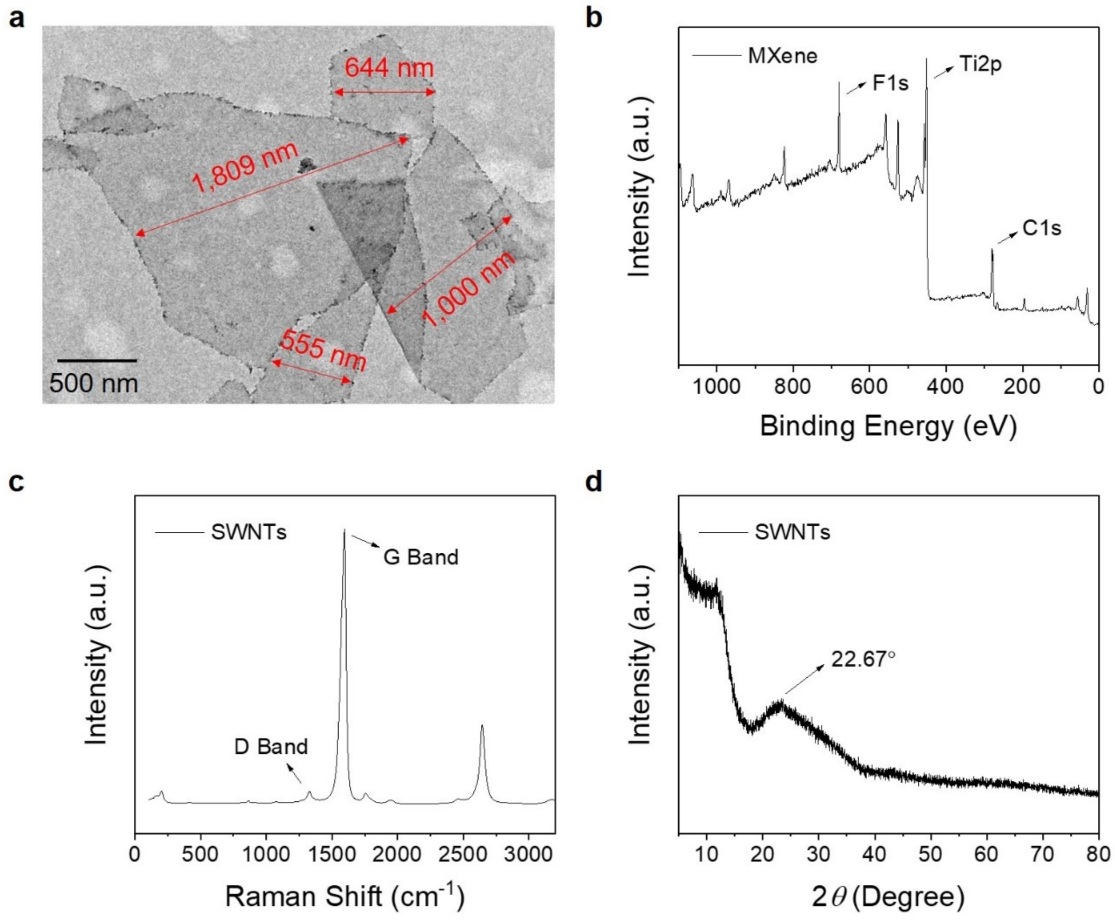

Supplementary Fig. 1 Characterization of $Ti_3C_2T_x$ MXene nanosheets and SWNTs. (**a**) Representative TEM image of $Ti_3C_2T_x$ MXene nanosheets. The average diameter of as-exfoliated MXene nanosheets was measured to be ca. 1,000 nm. (**b**) XPS spectrum of MXene nanosheets. The characteristic peaks of F1s, Ti2p, and C1s were observed. (**c**) Raman spectrum of SWNTs. Two representative D and G peaks at 1,324 and 1,596 cm^{-1} were observed, respectively. The I_D/I_G ratio was 0.049, indicating a low level of defects in SWNTs. (**d**) XRD pattern of SWNTs with a representative peak of 22.67°.

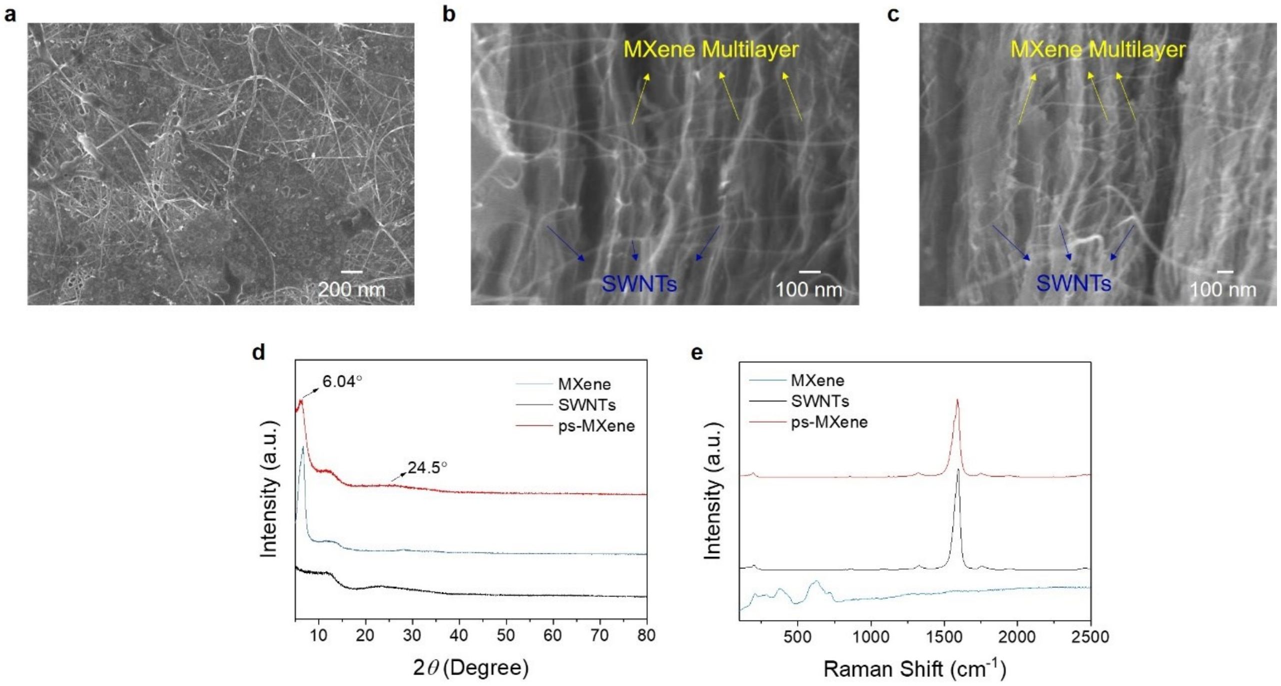

Supplementary Fig. 2 Characterizations of ps-MXene nanolayers. (**a**) Top-down SEM image of a ps-MXene nanolayer. The mass ratio of MXene/SWNT/PVA (wt.%) was set at 85/10/5. Interconnected SWNTs were observed on the surface of ps-MXene nanolayer. (**b**)(**c**) Cross-sectional SEM images of a ps-MXene nanolayer. SWNTs were well dispersed within the ps-MXene nanolayer. (**d**) XRD patterns of $Ti_3C_2T_x$ MXene, SWNTs, and ps-MXene nanolayer. The ps-MXene nanolayer exhibited the (002) diffraction peak of MXene multilayers at 6.04° and the representative peaks of SWNTs at 24.5°, indicating the prospective assembly of MXene nanosheets and SWNTs. (e) Raman spectra of MXene nanosheets, SWNTs, and ps-MXene nanolayer (at the MXene/SWNT/PVA ratio of 85/10/5).

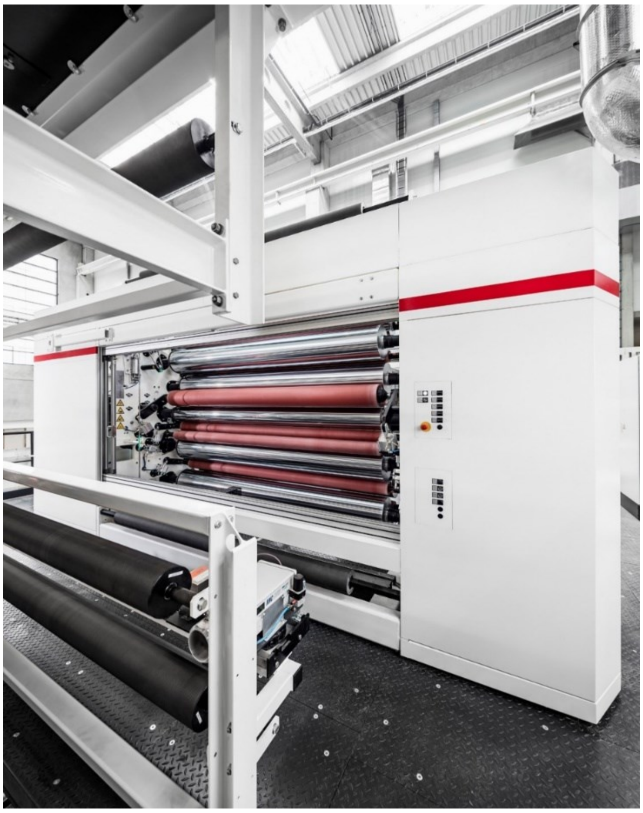

Supplementary Fig. 3 Machine for roll-to-roll production of uniaxial shrink films. Product of Windmöller & Hölscher.

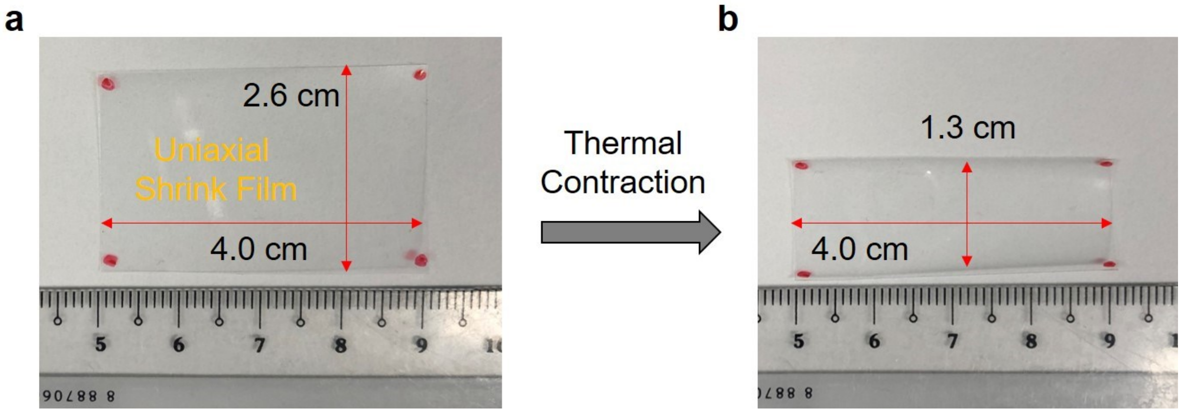

Supplementary Fig. 4 Thermal contraction of a uniaxial PS shrink film. (**a**) Photo of a uniaxial PS shrink film before thermal contraction. (**b**) Photo of a uniaxial PS shrink film after thermal contraction. The length remained at 4.0 cm, and the width was reduced from 2.6 to 1.3 cm, which was about 50% of the original width.

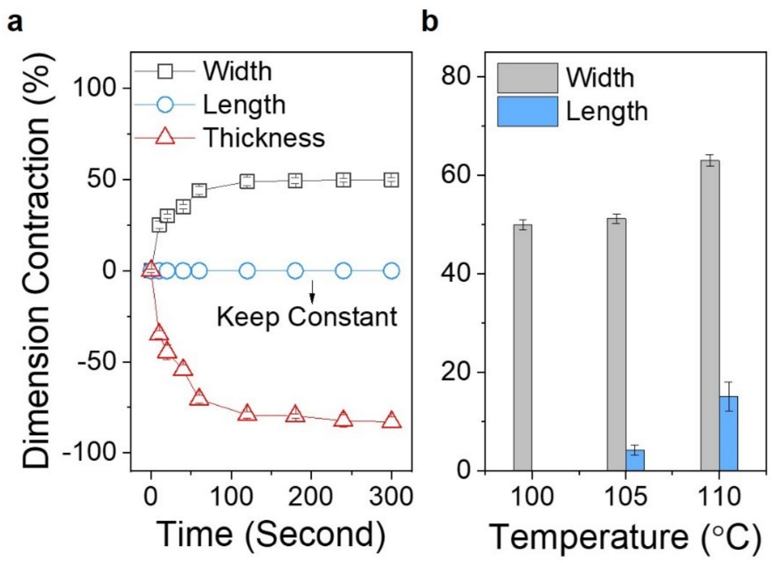

Supplementary Fig. 5 Dimension change of uniaxial shrink films after thermal contraction. (**a**) Dimension contraction of a uniaxial shrink film at 100 °C for different time lengths. (**b**) Dimension contraction of uniaxial shrink films at different temperatures for 120 seconds.

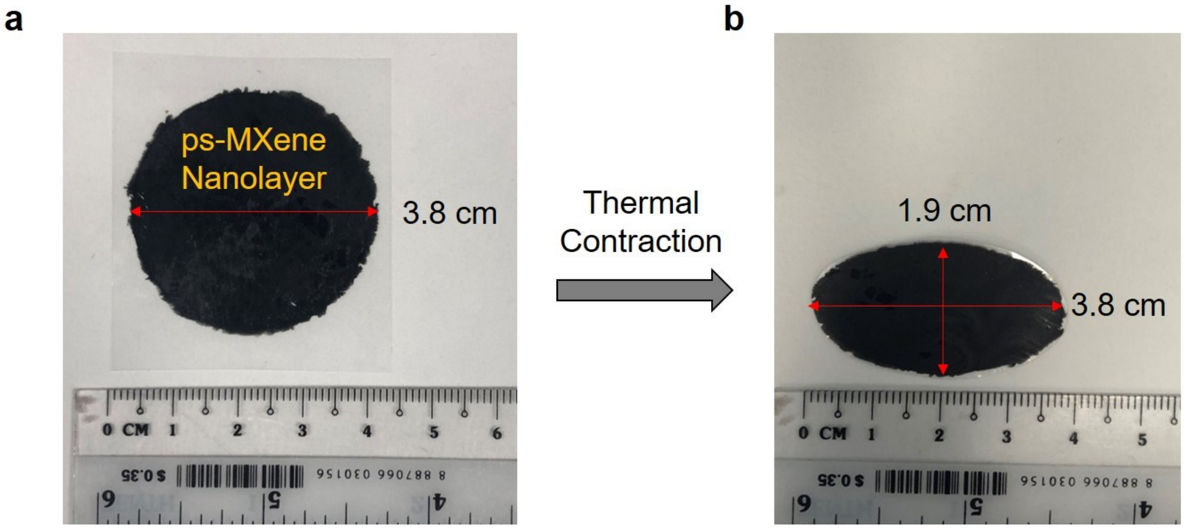

Supplementary Fig. 6 Uniaxial shrinkage of a ps-MXene nanolayer. (**a**) Photo of a ps-MXene nanolayer before uniaxial shrinkage. (**b**) Photo of a ps-MXene nanolayer after uniaxial shrinkage. The length remained at 3.8 cm, while the width was reduced from 3.8 to 1.9 cm, which was about 50% of the original width.

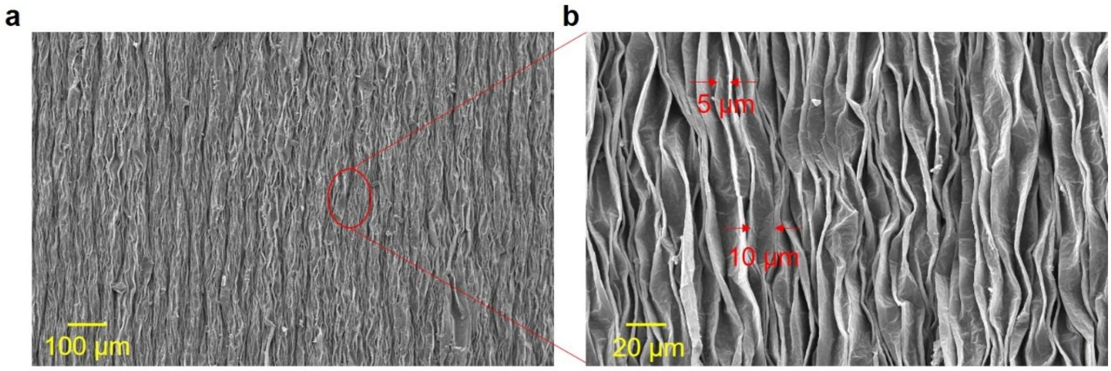

Supplementary Fig. 7 (a) SEM image and (b) high-resolution SEM image of a M_w nanolayer.

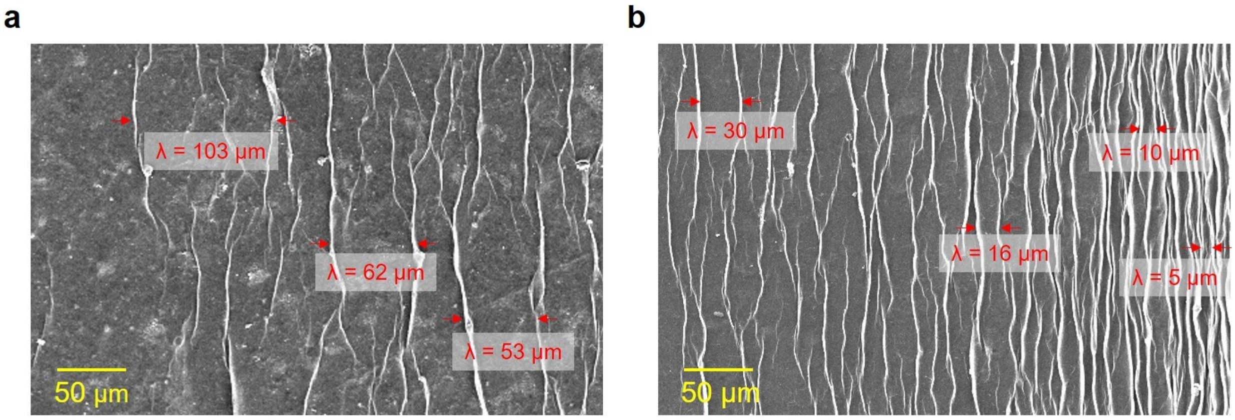

Supplementary Fig. 8 High-resolution SEM images of the transition zone of a $M_{p\text{-}w\text{-}p}$ nanolayer, where the wrinkle wavelength gradually increased.

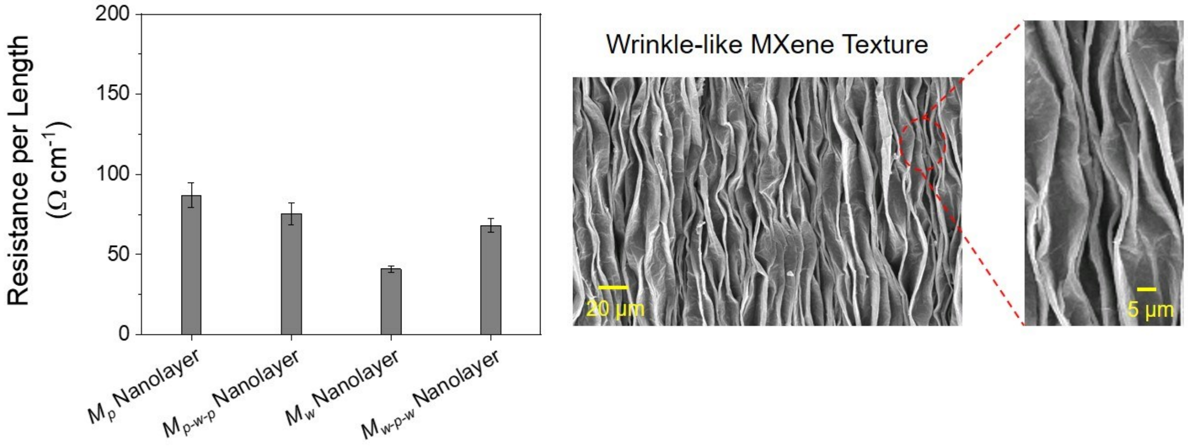

Supplementary Fig. 9 Electrical resistances of all kinds of M_n nanolayers. The lower resistances of $M_{p\text{-}w\text{-}p}$, M_w, and $M_{w\text{-}p\text{-}w}$ nanolayers were attributed to the peak contacts between dense wrinkles, which shortened the electrical pathways. The width of all M_n nanolayers was controlled to be 1 cm and the MXene/SWNT/PVA ratio was controlled as 85/10/5.

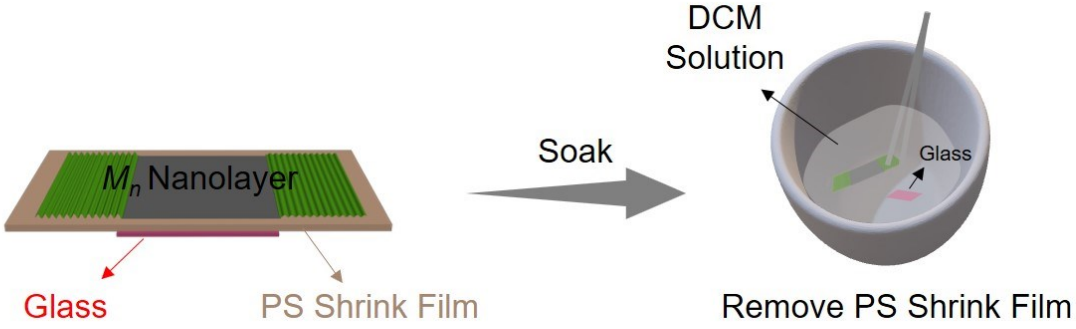

Supplementary Fig. 10 Fabrication of freestanding M_n nanolayer.

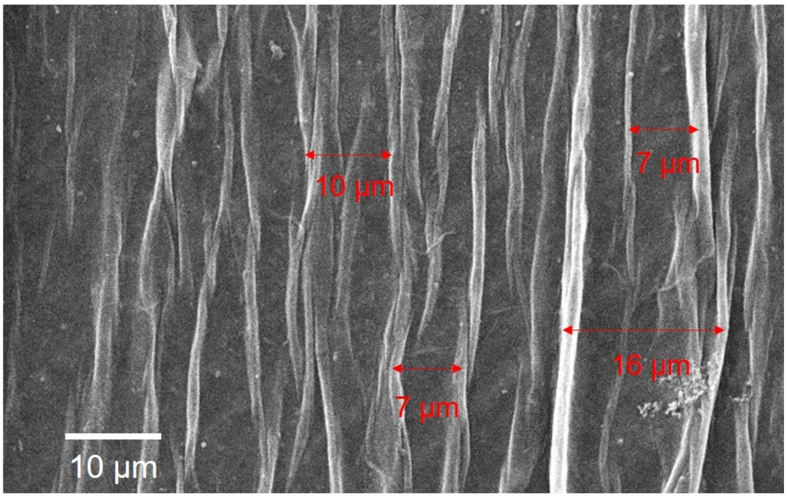

Supplementary Fig. 11 SEM image of an as-transferred M_w nanolayer. The average wrinkle wavelength was ca. 10.2 µm.

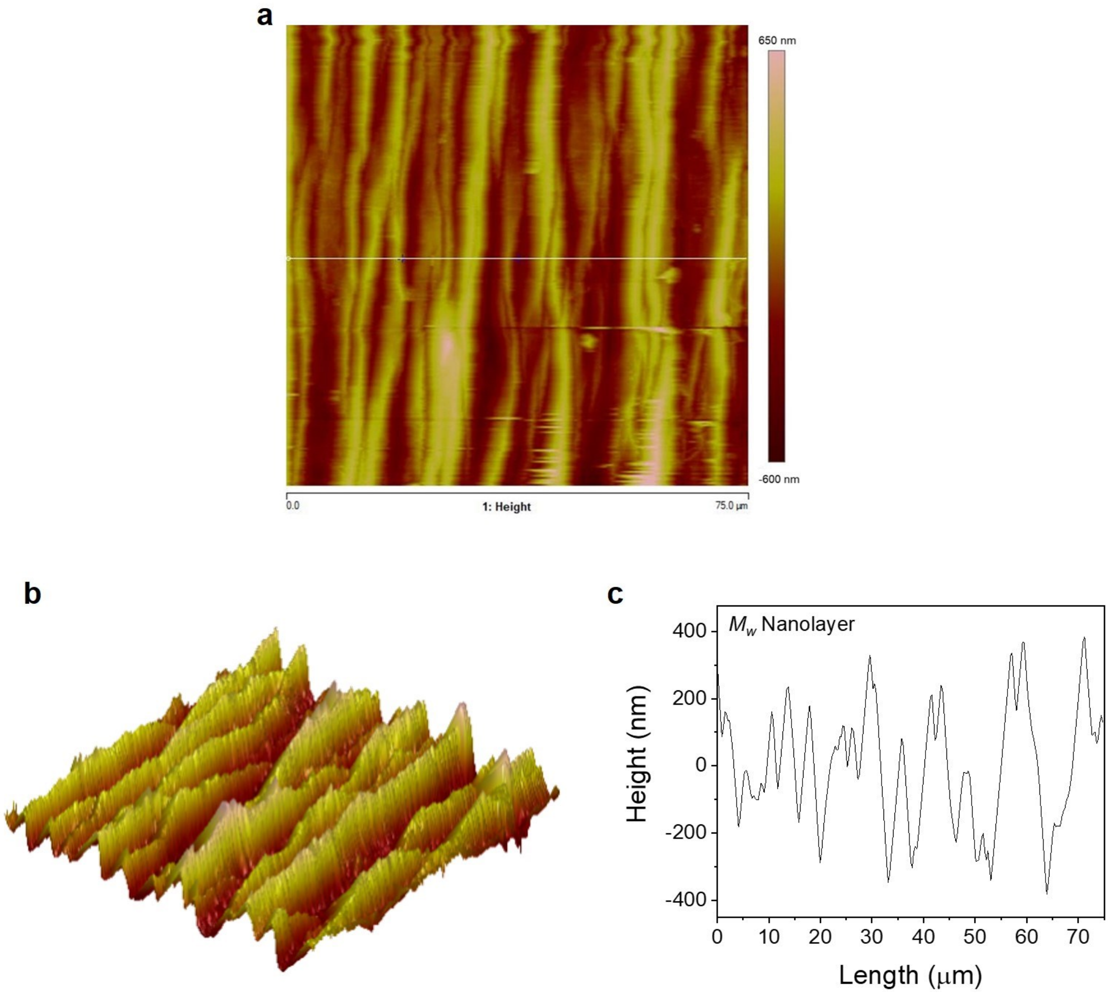

Supplementary Fig. 12 AFM characterizations of M_w nanolayers. (**a**) AFM image of a M_w nanolayer. (**b**) 3D AFM image of a M_w nanolayer. (**c**) Depth profile of a M_w nanolayer along the white line in (**a**).

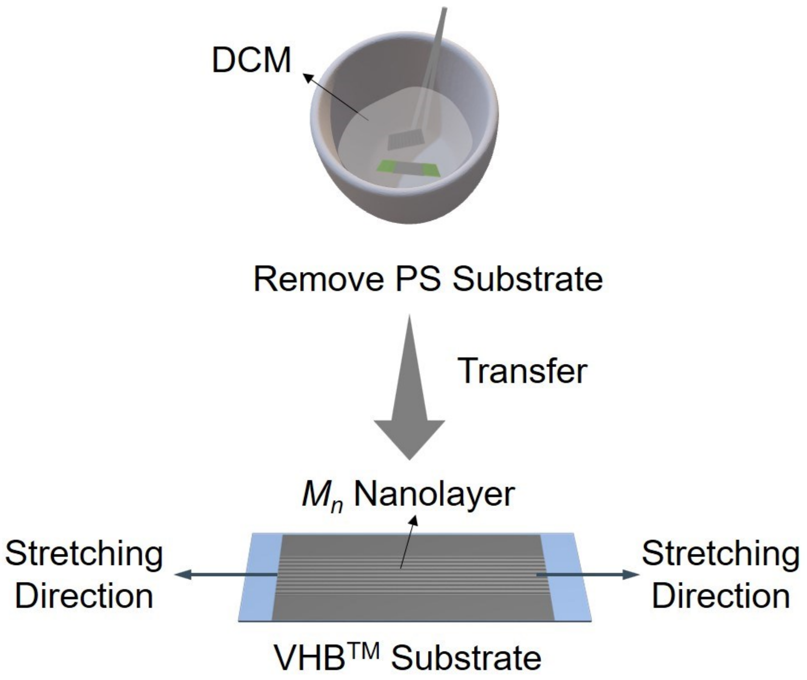

Supplementary Fig. 13 Schematic illustration of transferring a freestanding ps-MXene nanolayer onto a VHB^{TM} substrate.

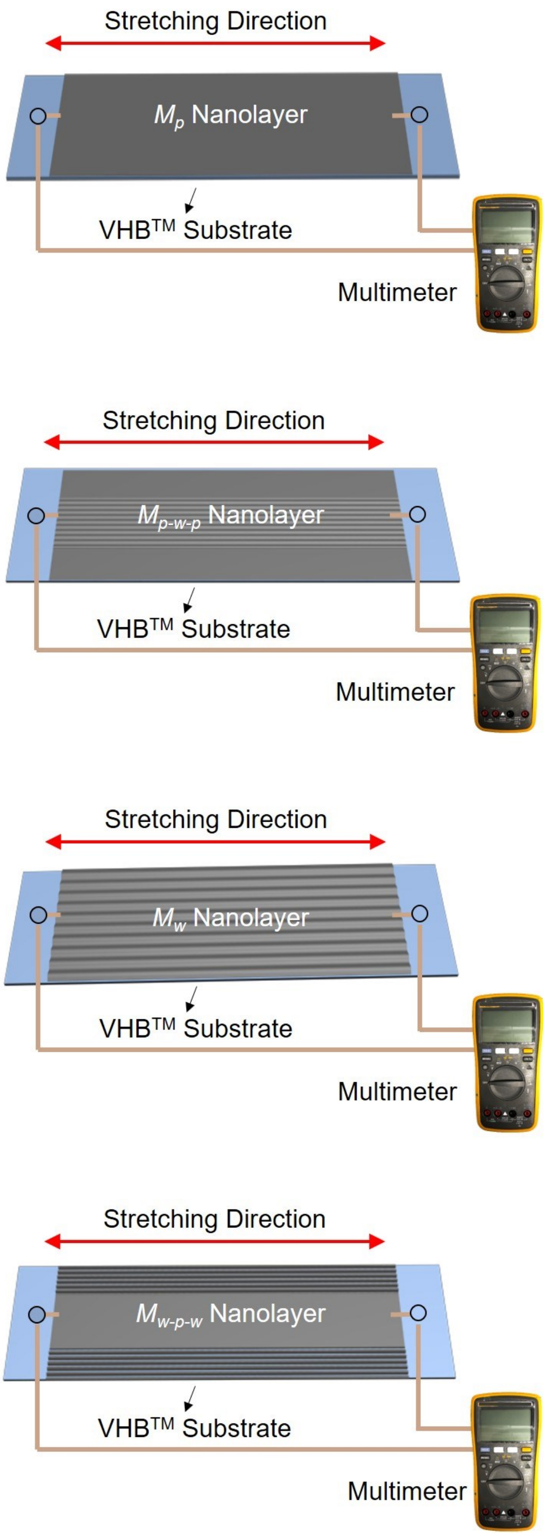

Supplementary Fig. 14 Fabrication of M_n sensors. Copper wires were connected to the two ends of a M_n nanolayer. By applying uniaxial strains on the M_n sensor along the axes of uniaxial wrinkles (not perpendicular to the axes of wrinkles), its relative resistance change (S_ε)–strain (ε) profile was monitored and recorded by a multimeter.

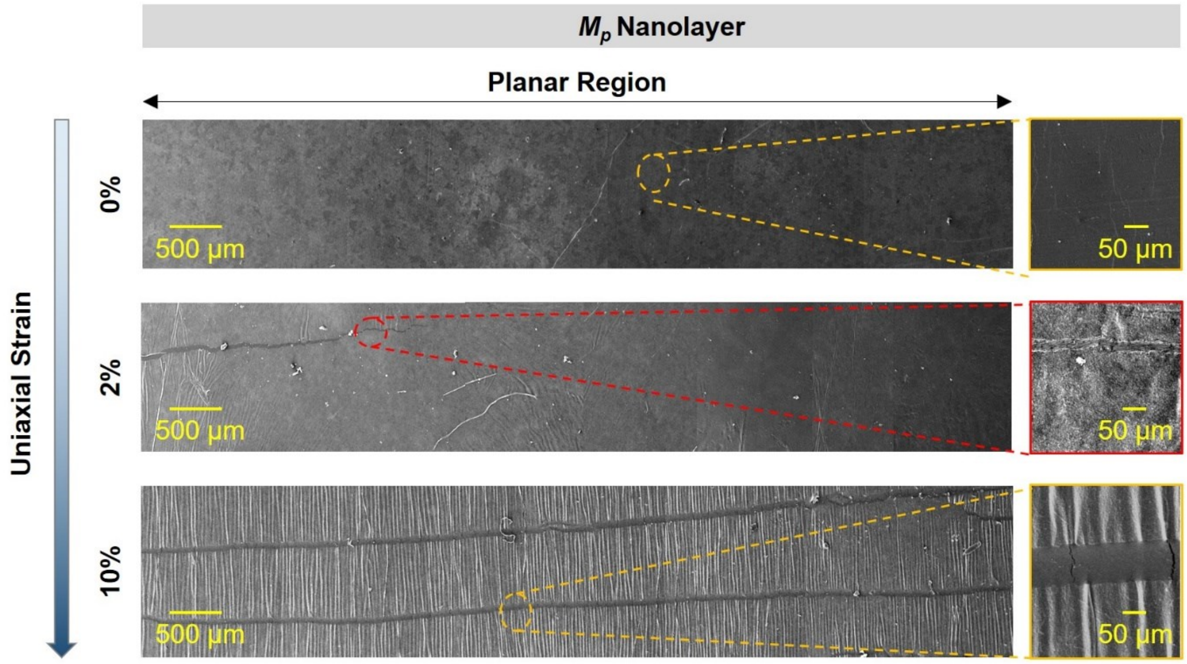

Supplementary Fig. 15 SEM images of a M_p nanolayer under uniaxial strains. Under uniaxial strains, long and continuous fractures emerged on the M_p nanolayer.

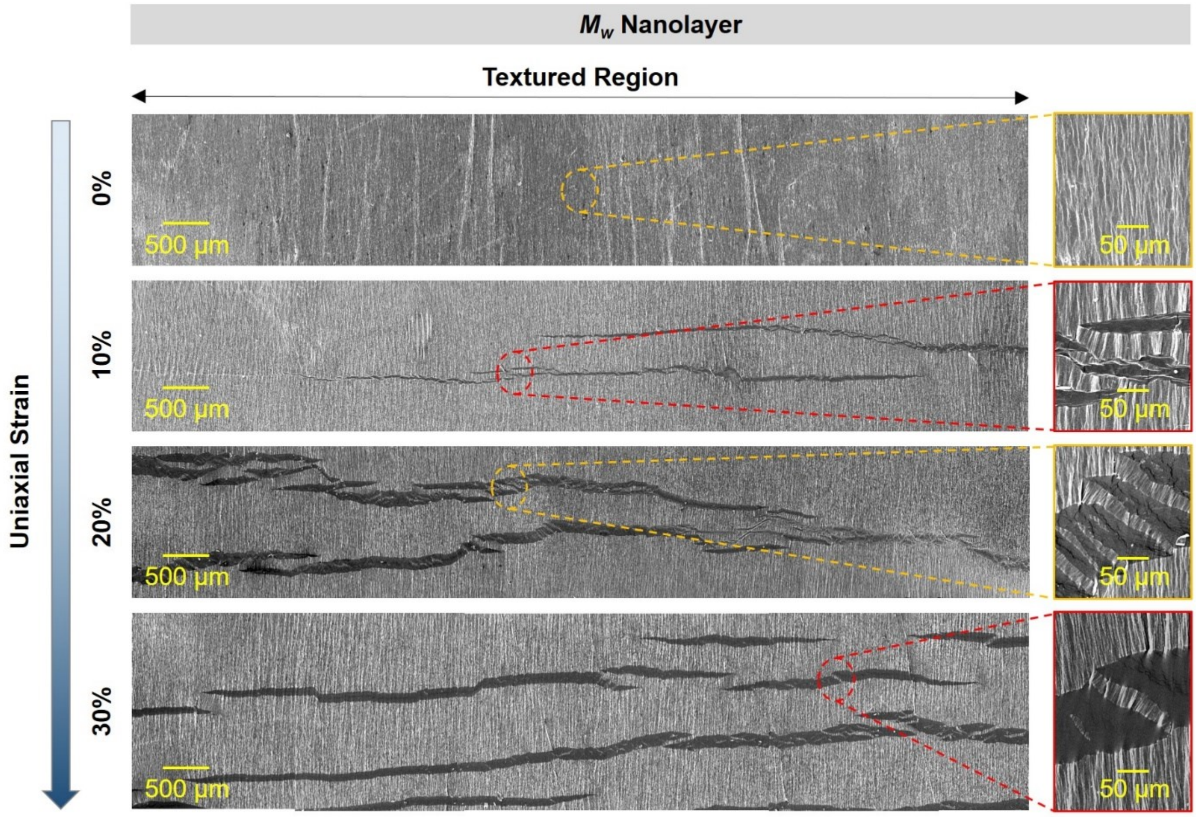

Supplementary Fig. 16 SEM images of a M_w nanolayer under uniaxial strains. Under uniaxial strains, short and zigzag cracks gradually propagated.

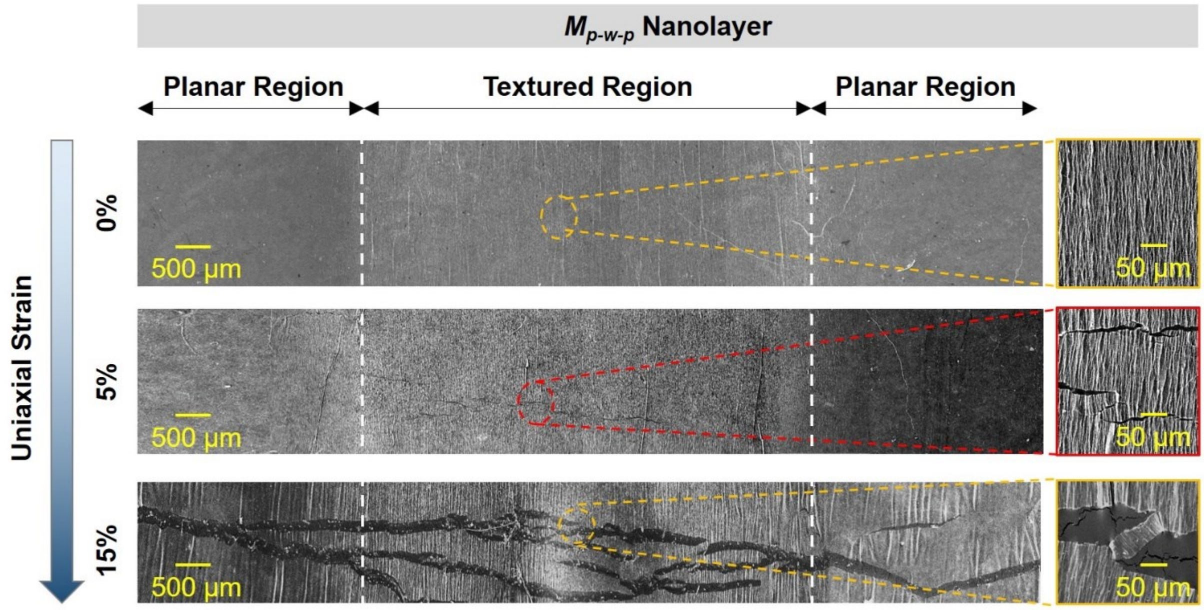

Supplementary Fig. 17 SEM images of a $M_{p\text{-}w\text{-}p}$ nanolayer under uniaxial strains. Under uniaxial strains, short and zigzag cracks gradually propagated in the middle region, while long fractures were developed at the two ends.

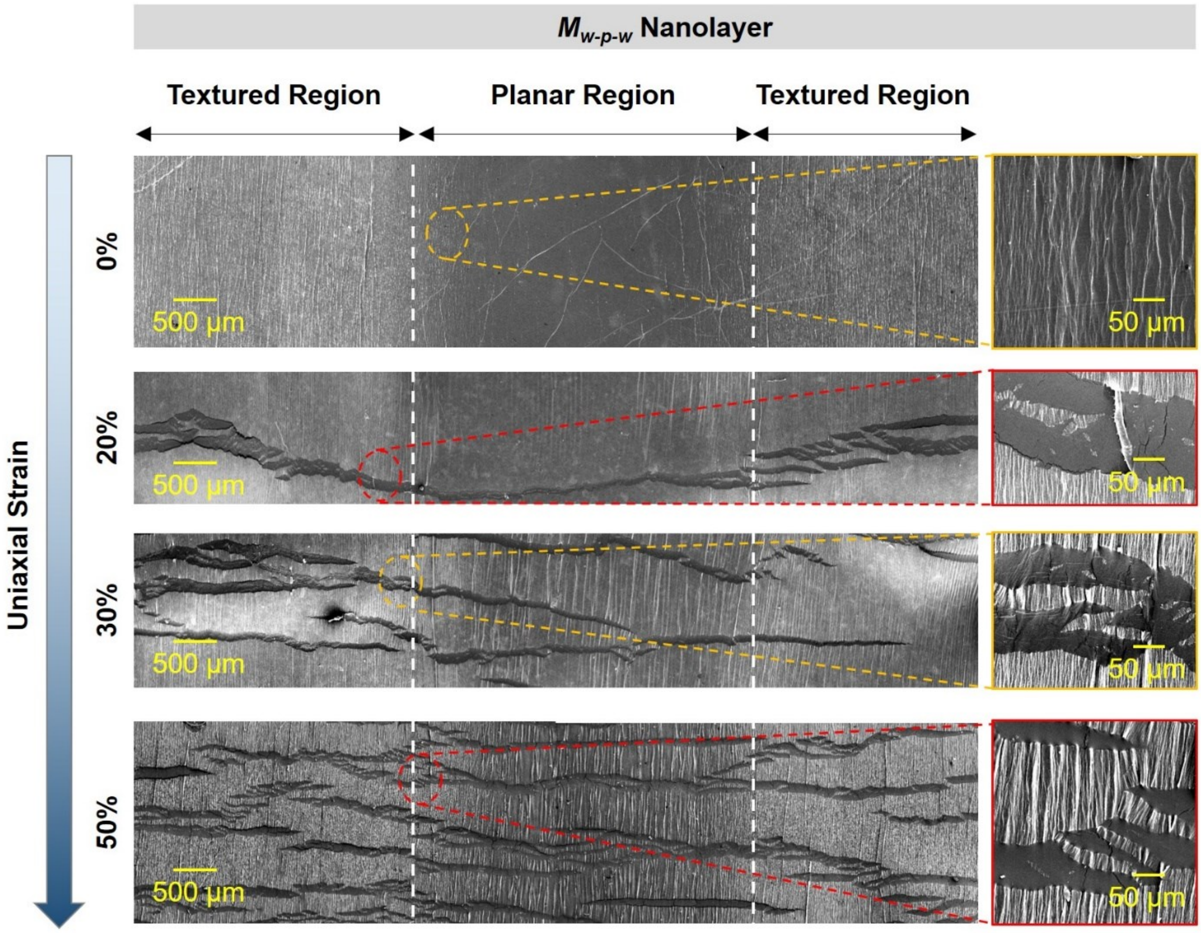

Supplementary Fig. 18 SEM images of a M_{w-p-w} nanolayer under uniaxial strains. Wrinkle-like microtextures were shown at the two ends of a M_{w-p-w} nanolayer. Under uniaxial stretching, short and zigzag cracks gradually propagated at the two ends, while long fractures were developed in the middle region.

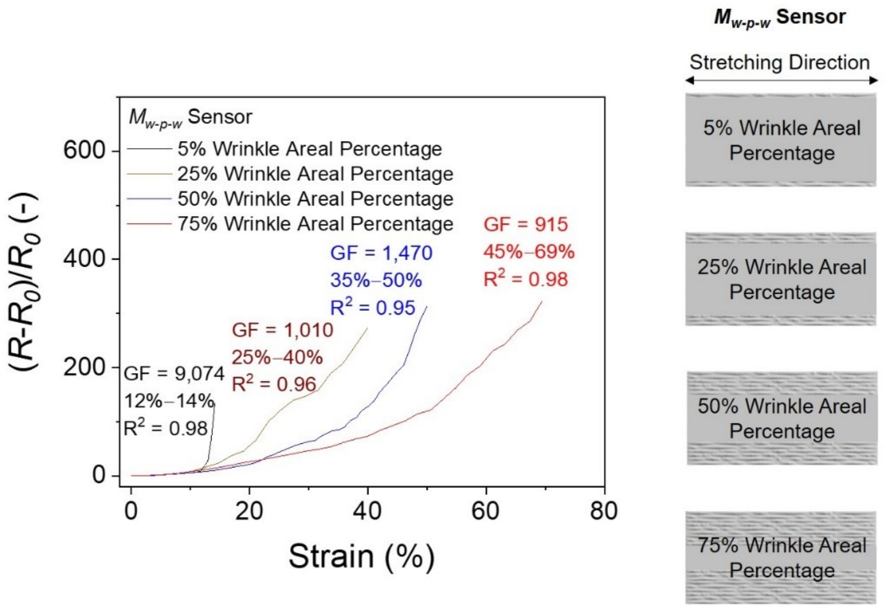

Supplementary Fig. 19 Strain sensing curves of *M_{w-p-w}* sensors with areal percentages of wrinkle-like region(s) from 5% to 75%.

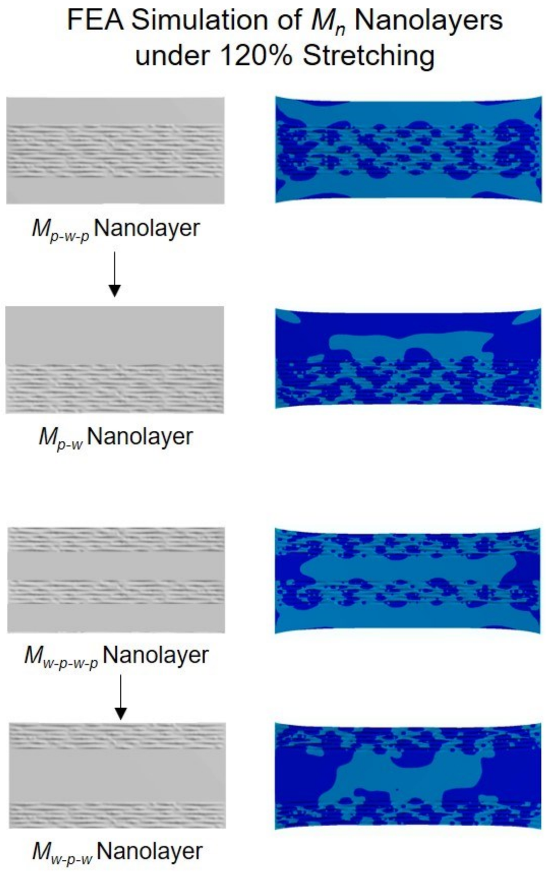

Supplementary Fig. 20 FEA simulation of four M_n nanolayers under 120% stretching.

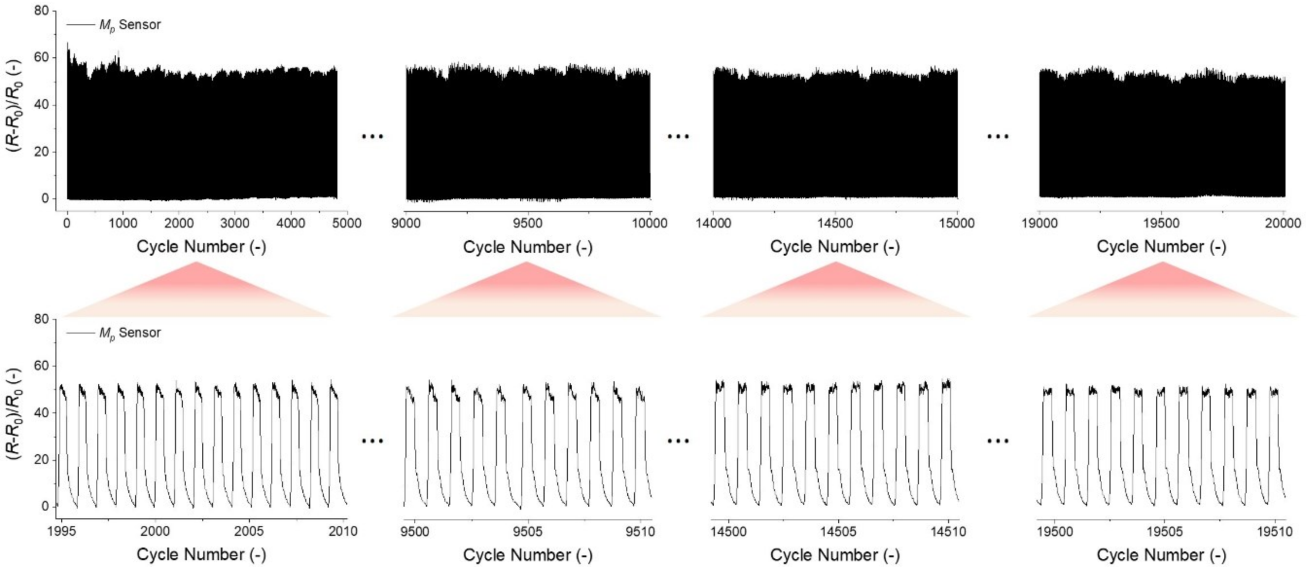

Supplementary Fig. 21 Cycling test of a M_p sensor under 5% strain for 20,000 cycles.

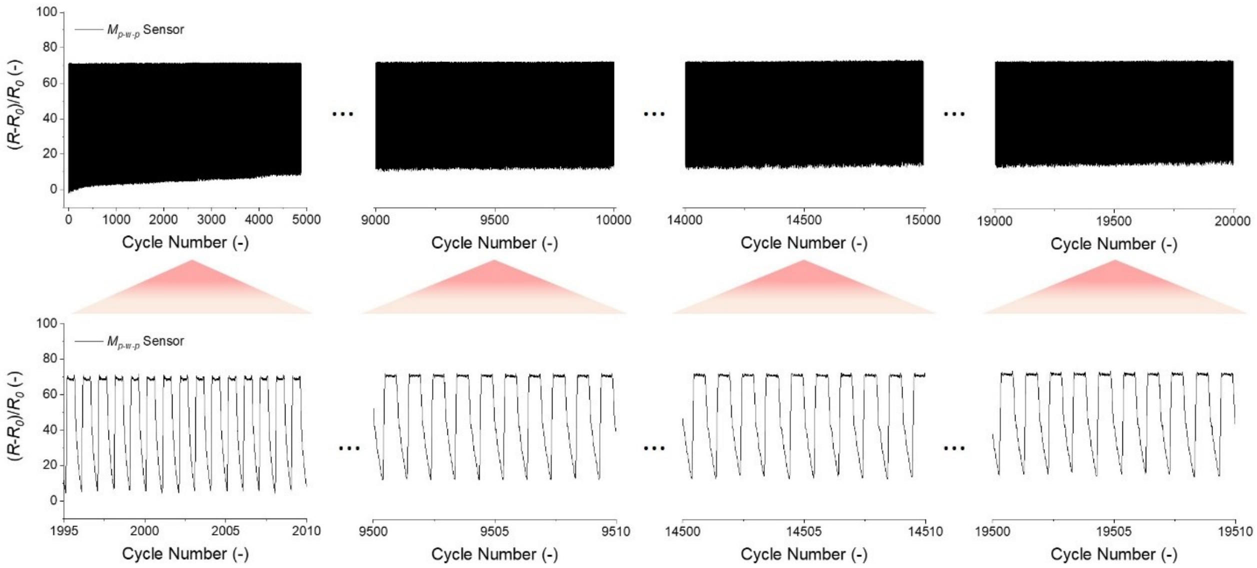

Supplementary Fig. 22 Cycling test of a *$M_{p\text{-}w\text{-}p}$* sensor under 15% strain for 20,000 cycles.

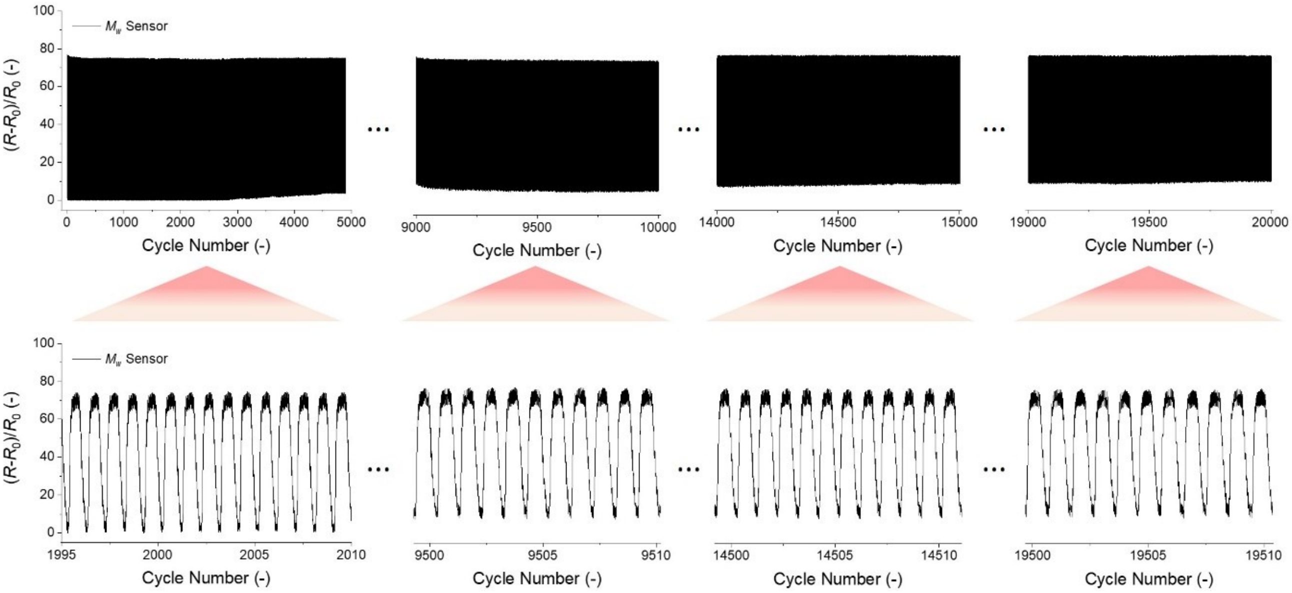

Supplementary Fig. 23 Cycling test of a M_w sensor under 25% strain for 20,000 cycles.

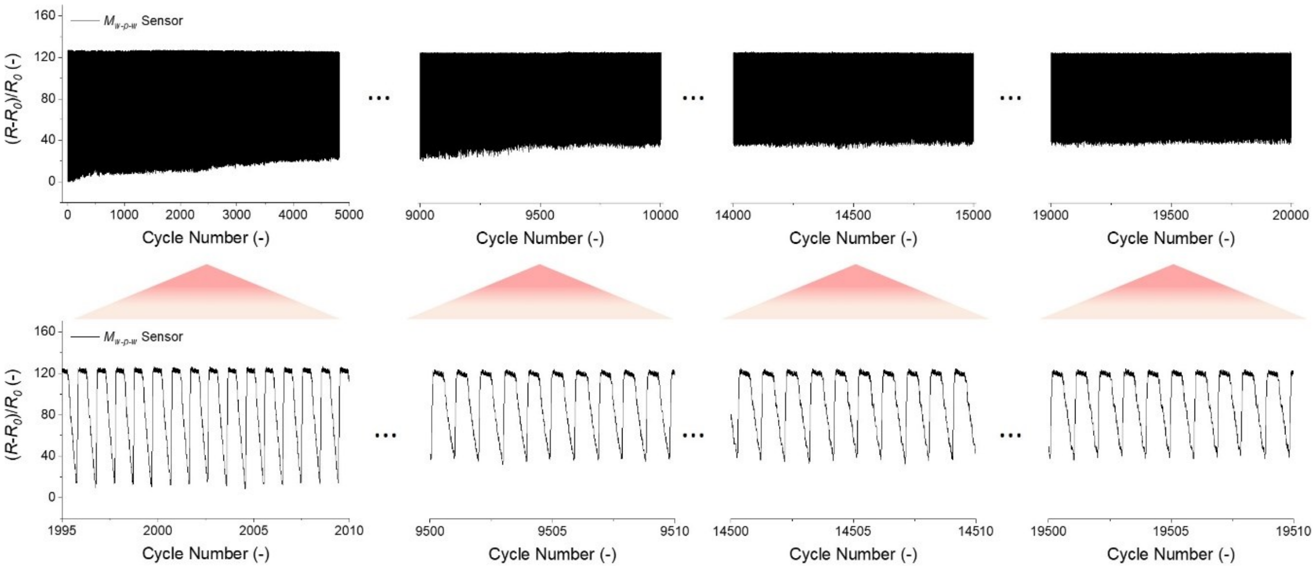

Supplementary Fig. 24 Cycling test of a *$M_{w\text{-}p\text{-}w}$* sensor under 40% strain for 20,000 cycles.

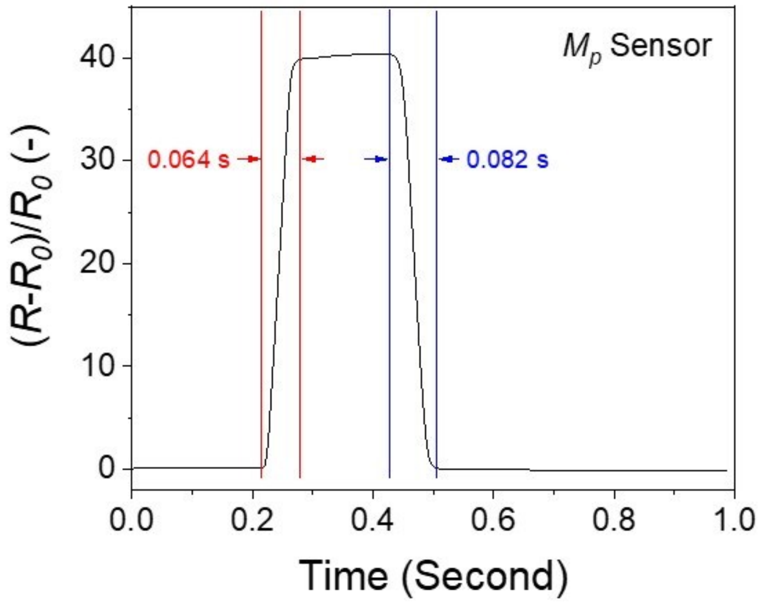

Supplementary Fig. 25 Response times of a M_p sensor in the stretching and relaxation processes. The response times of a M_p sensor in its stretching and relaxation processes were characterized to be 0.064 and 0.082 seconds, respectively.

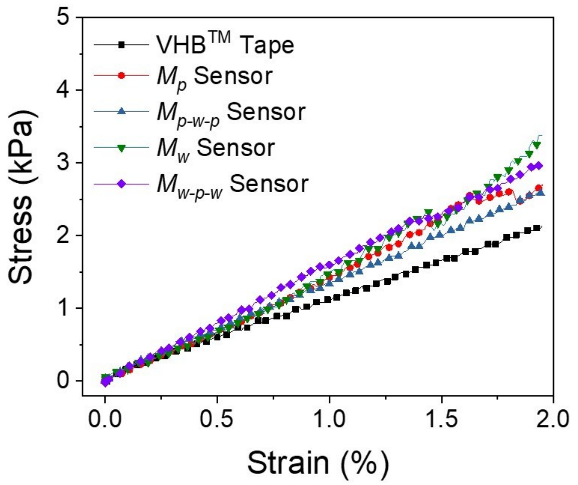

Supplementary Fig. 26 Stress–strain curves of all M_n sensors. As the nanolayer thickness was controlled at 400 nm, all M_p, $M_{p\text{-}w\text{-}p}$, M_w, and $M_{w\text{-}p\text{-}w}$ sensors showed similar Young's moduli of ca. 150 kPa, which were higher than a bare VHB™ tape (106 kPa).

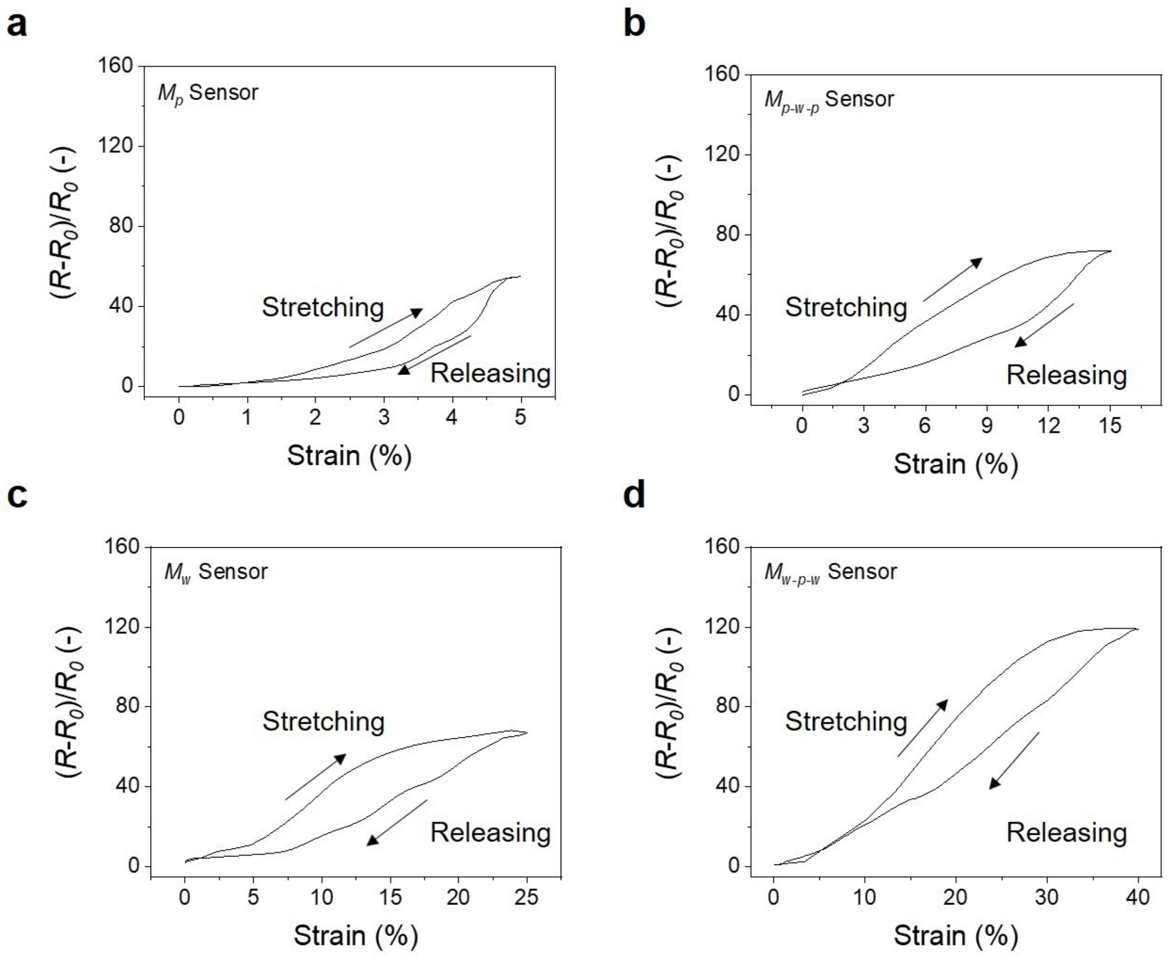

Supplementary Fig. 27 Hysteresis curves of M_n sensors.

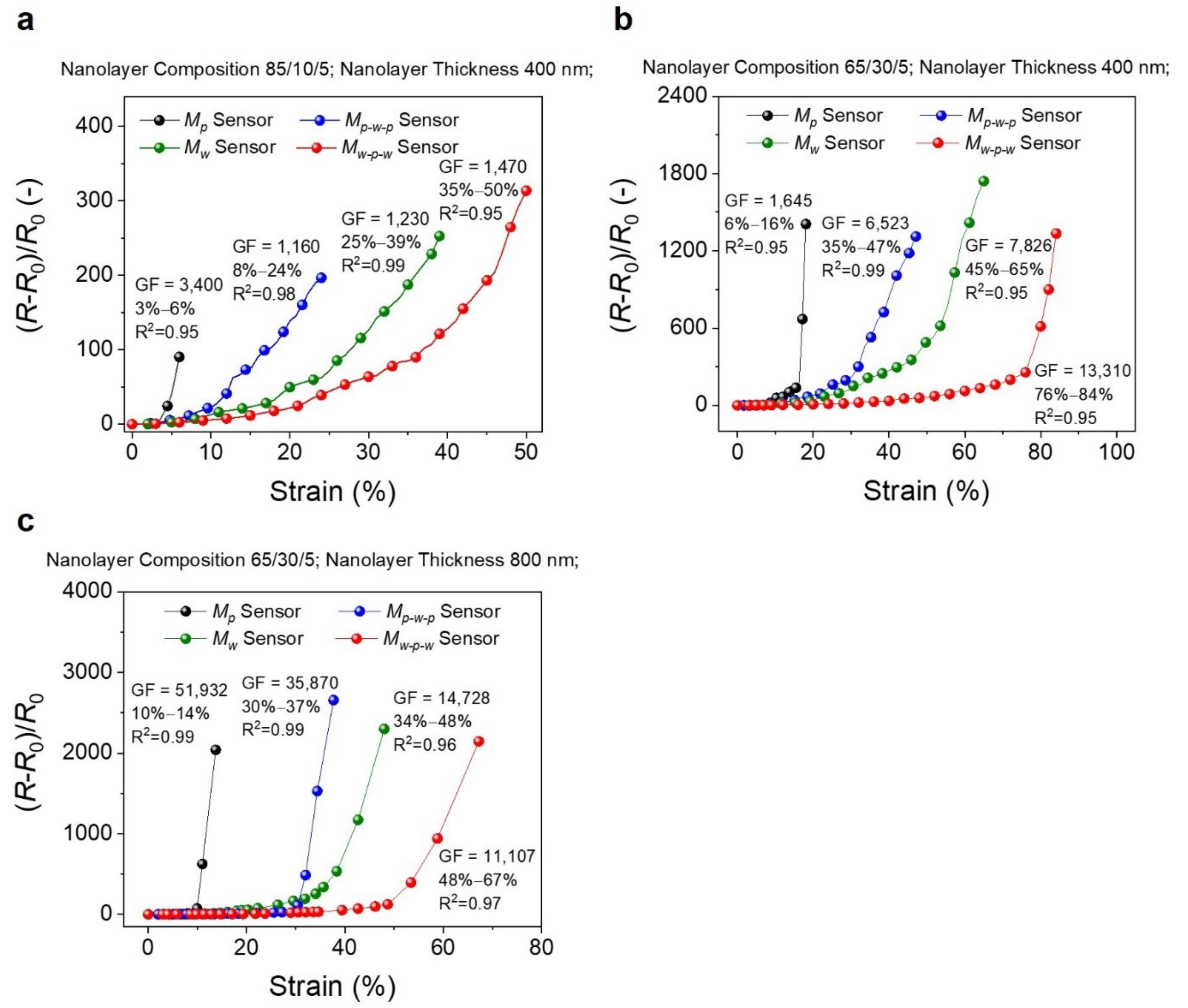

Supplementary Fig. 28 Signal outputs, S_ε, of M_n sensors with varying nanolayer compositions and nanolayer thicknesses under uniaxial strains. (**a**) The composition of all M_n nanolayers was set at 85/10/5 (MXene/SWNT/PVA), and the thickness of all M_n nanolayers was controlled at 400 nm. (**b**) The composition of all M_n nanolayers was set at 65/30/5 (MXene/SWNT/PVA), and the thickness of all M_n nanolayers was controlled at 400 nm. (**c**) The composition of all M_n nanolayers was set at 65/30/5 (MXene/SWNT/PVA), and the thickness of all M_n nanolayers was controlled at 800 nm.

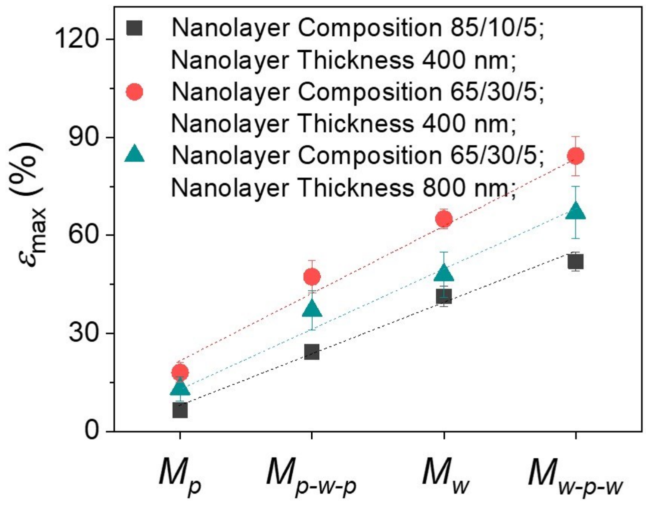

Supplementary Fig. 29 Nanolayer composition, thickness, and topography effects on the ε_{max} of M_n sensors.

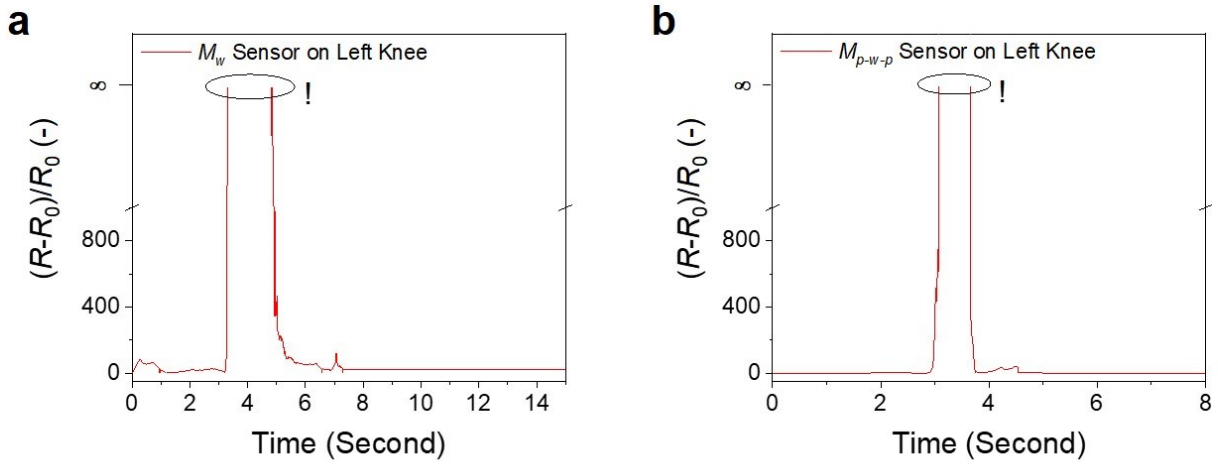

Supplementary Fig. 30 Signal outputs, S_ε, of (a) M_w sensor attached on the left knee, and (b) $M_{p\text{-}w\text{-}p}$ sensor attached on the left knee, during repeated squatting movements. Symbol "!" indicates that the M_n sensors' resistances increased to infinite, where M_n sensors lost their strain sensing capabilities.

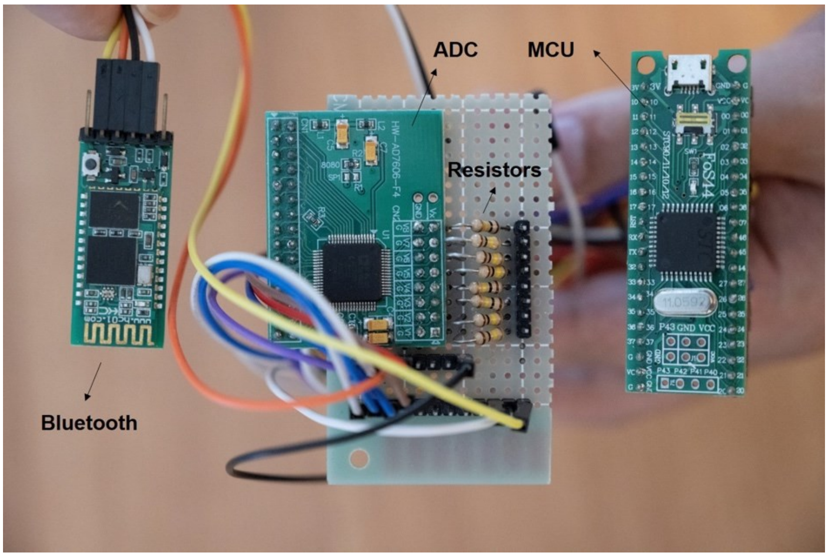

Supplementary Fig. 31 Circuit design of a wireless sensor module, consisting of seven M_n sensors, an ADC unit, a MCU unit, seven standard resistors, and a Bluetooth chip.

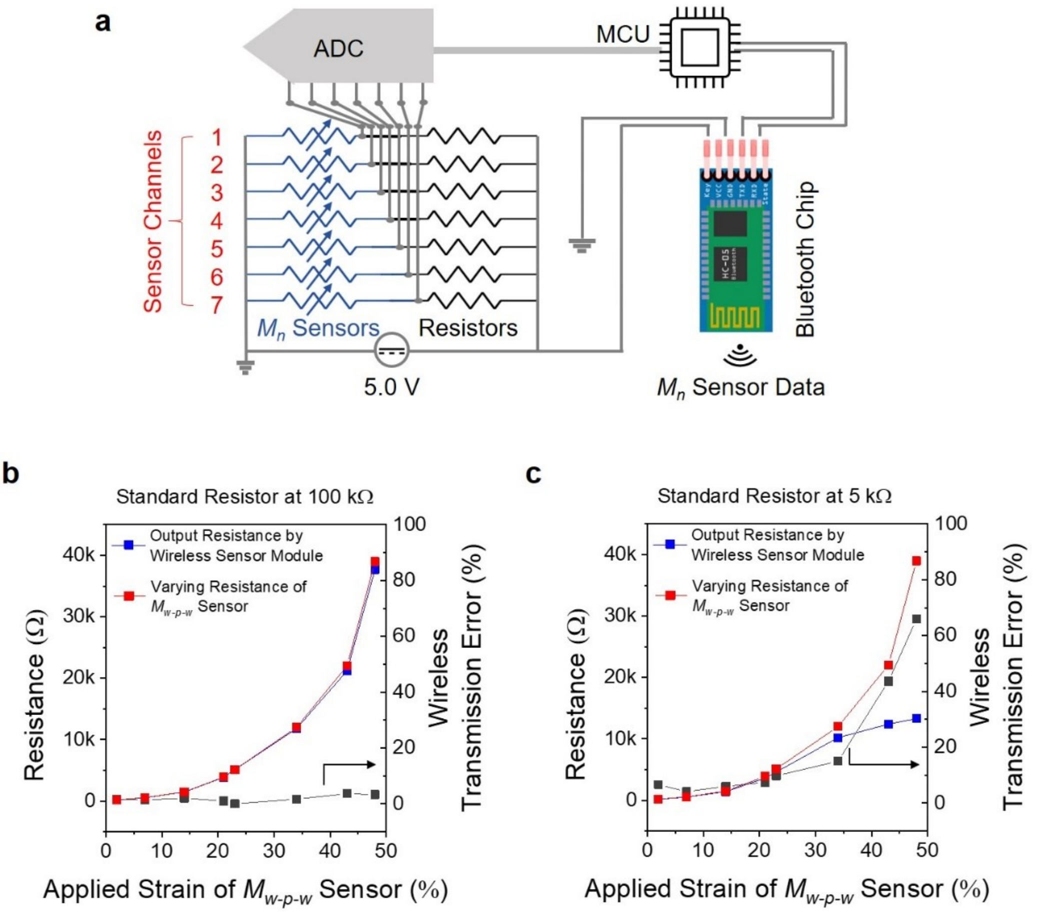

Supplementary Fig. 32 Wireless transmission errors of wireless sensor modules. (a) Circuit of a wireless sensor module. (**b**) Low transmission errors (average errors <4%) were observed in the wireless sensor module with 100-kΩ standard resistors. (**c**) High transmission errors (average errors >20%) were observed in the wireless sensor module with 5-kΩ standard resistors. Here, the wireless transmission error was measured by using a $M_{w\text{-}p\text{-}w}$ sensor under different strains.

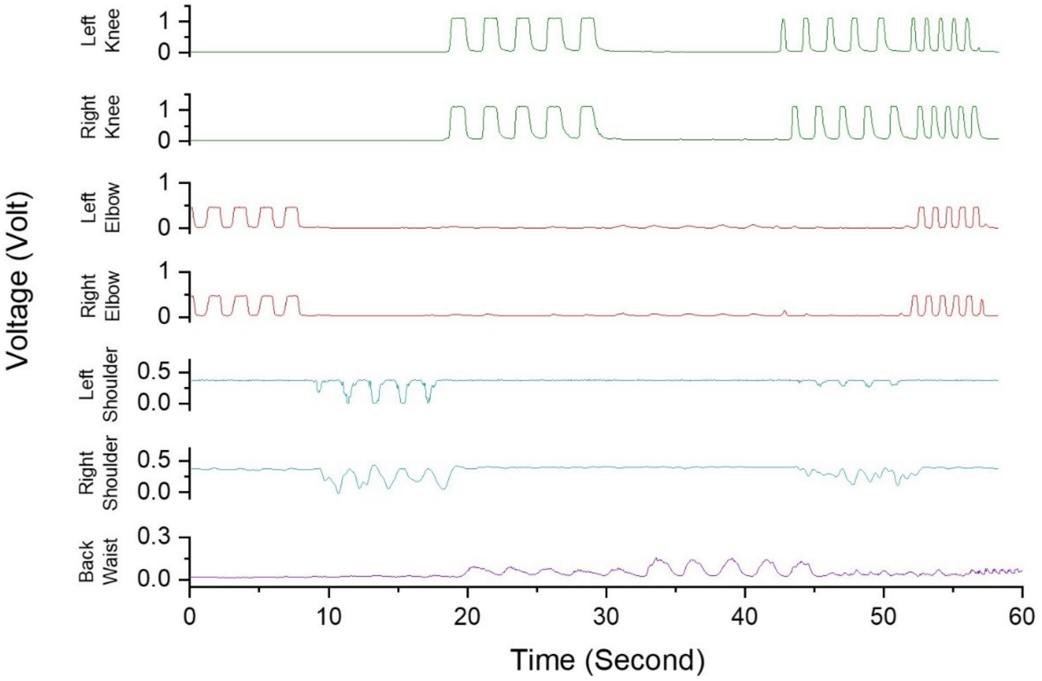

Supplementary Fig. 33 Voltage signals of wireless sensor module from seven M_n sensors during full-body motion monitoring, including (i) left/right elbow lifting, (ii) left/right shoulder lifting, (iii) squatting, (iv) stooping, (v) walking, and (vi) running.

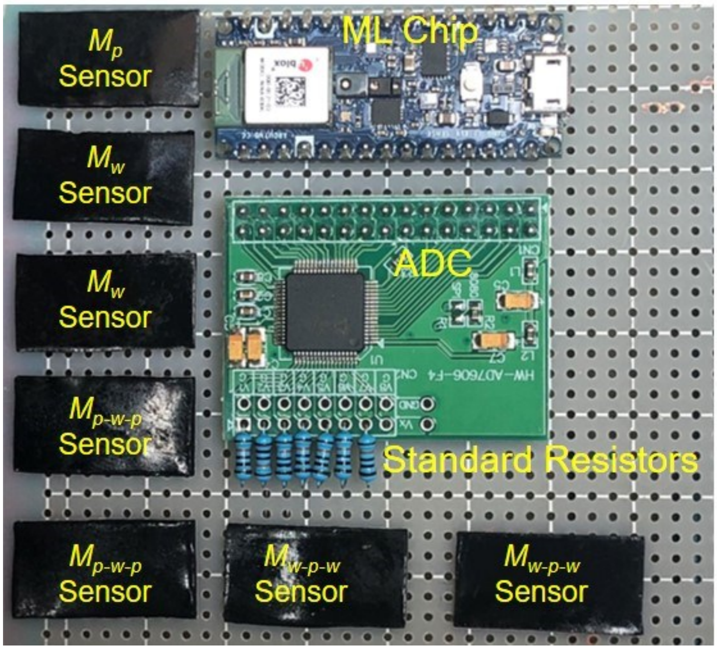

Supplementary Fig. 34 Circuit design of an edge sensor module, consisting of seven M_n sensors, an ADC unit, seven standard resistors, and an ARDUINO chip.

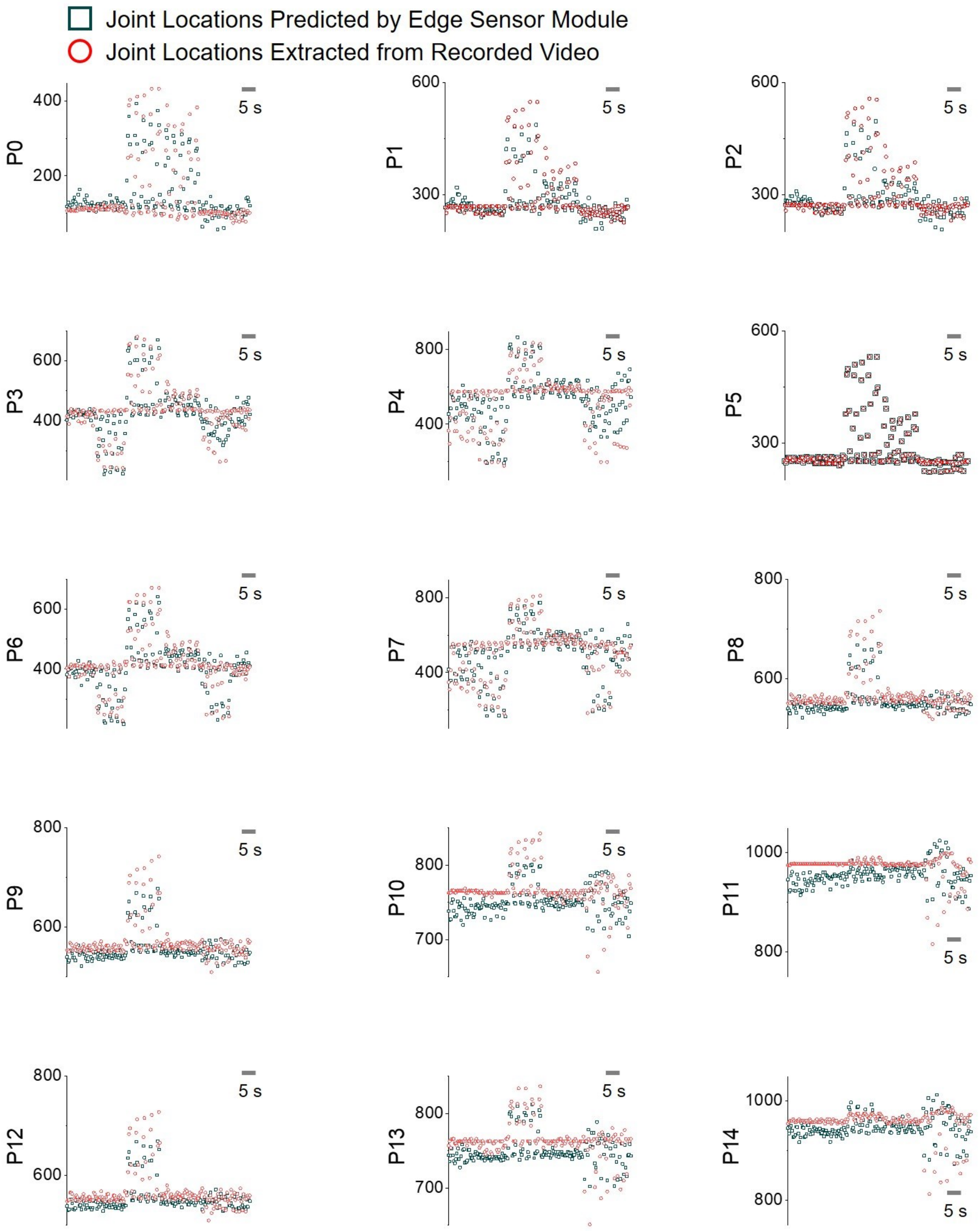

Supplementary Fig. 35 Comparison between 15 joint locations (from P0 to P14) determined by an edge sensor module and extracted from recorded video.

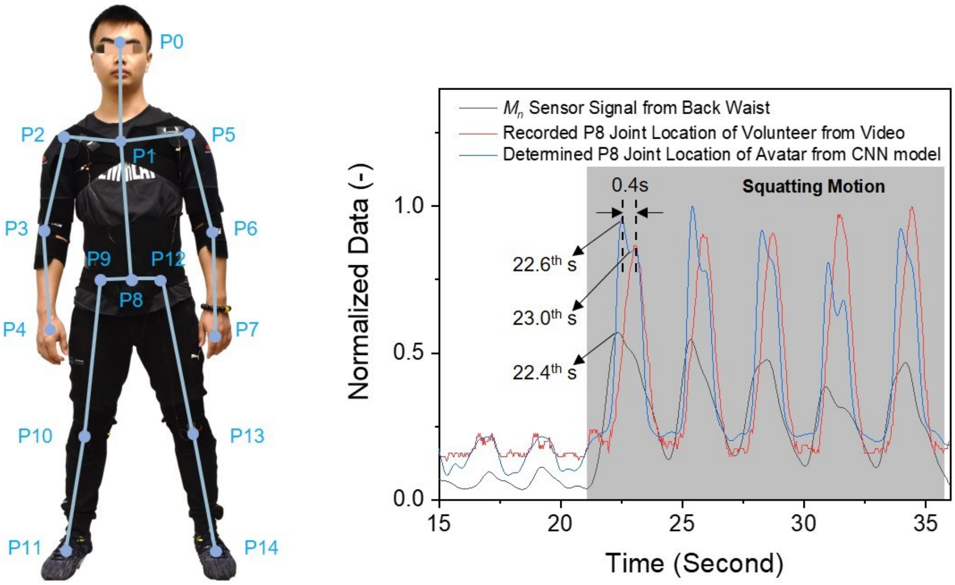

Supplementary Fig. 36 Signal timelines of a M_p sensor on the back waist and the location trajectories of P8 joint that were extracted from the recorded video or determined from the CNN model.

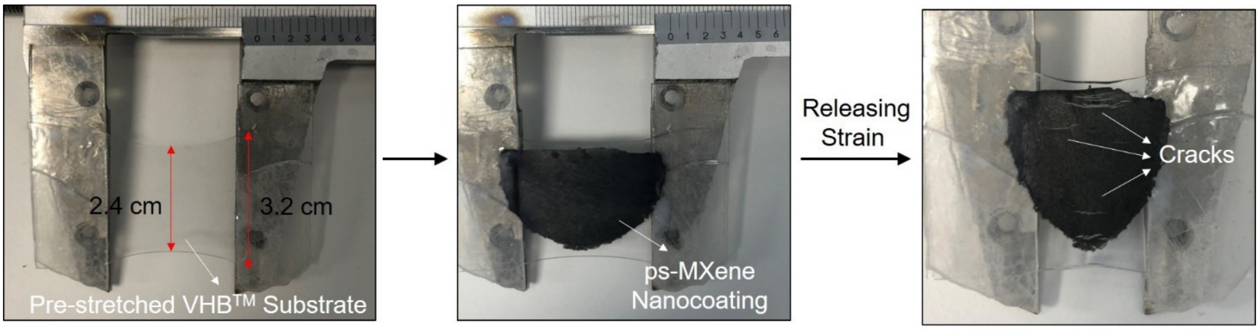

Supplementary Fig. 37 Fabrication of wrinkle-like microtextures by using pre-stretched VHB™ substrates. First, a M_p nanolayer was transferred onto a pre-stretched VHB™ substrate. After the strain released, periodic wrinkles were generated. However, visible cracks were observed on the deformed ps-MXene nanolayer due to the large Poisson's ratio of VHB™ tapes (0.5), leading to unfavorable orthogonal stretching during the strain relaxation process.

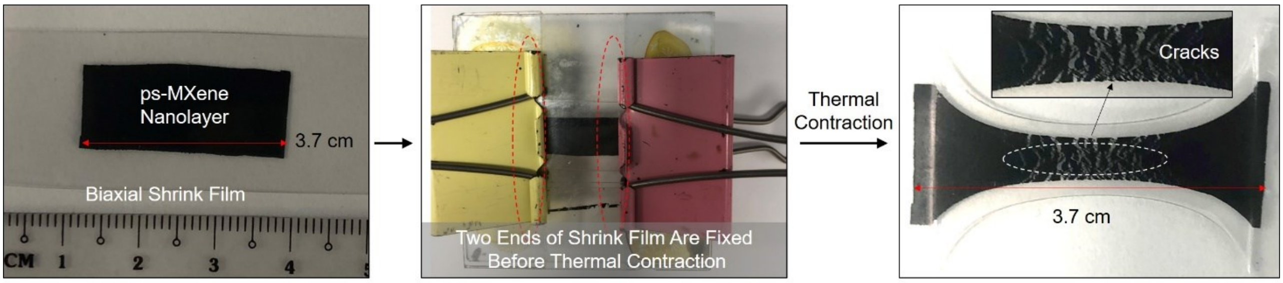

Supplementary Fig. 38 Fabrication of wrinkle-like microtextures by using biaxial shrink films. First, a M_p nanolayer was transferred onto a biaxial shrink film. The two ends of M_p nanolayer-coated shrink film were fixed during thermal contraction. After the uniaxial strain was released, periodic wrinkles were generated. However, visible cracks were observed due to large orthogonal compression.

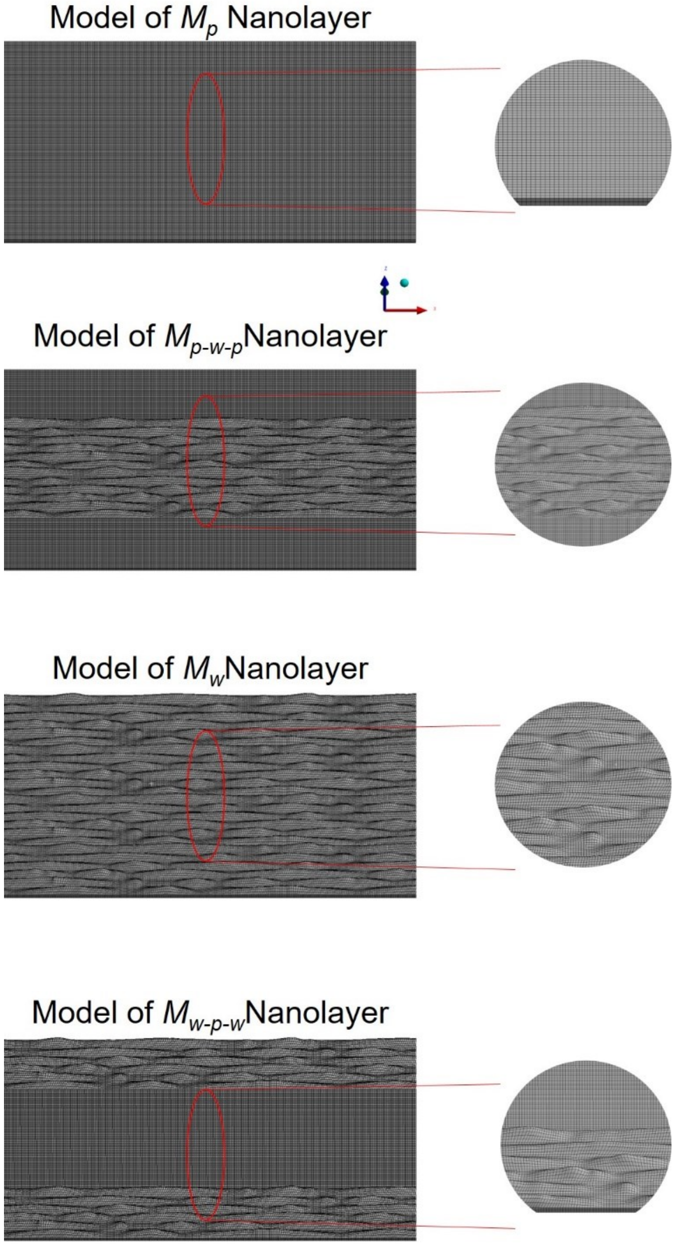

Supplementary Fig. 39 FEA models of M_n microstructures. FEA models were built by the Static Structural module of ANSYS Workbench 19.0. The simulation parameters were set for ps-MXene nanolayers as follows: Young's modulus of 1.7 GPa, Poisson's ratio of 0.227, and mass density of 1.25g cm^{-3}.

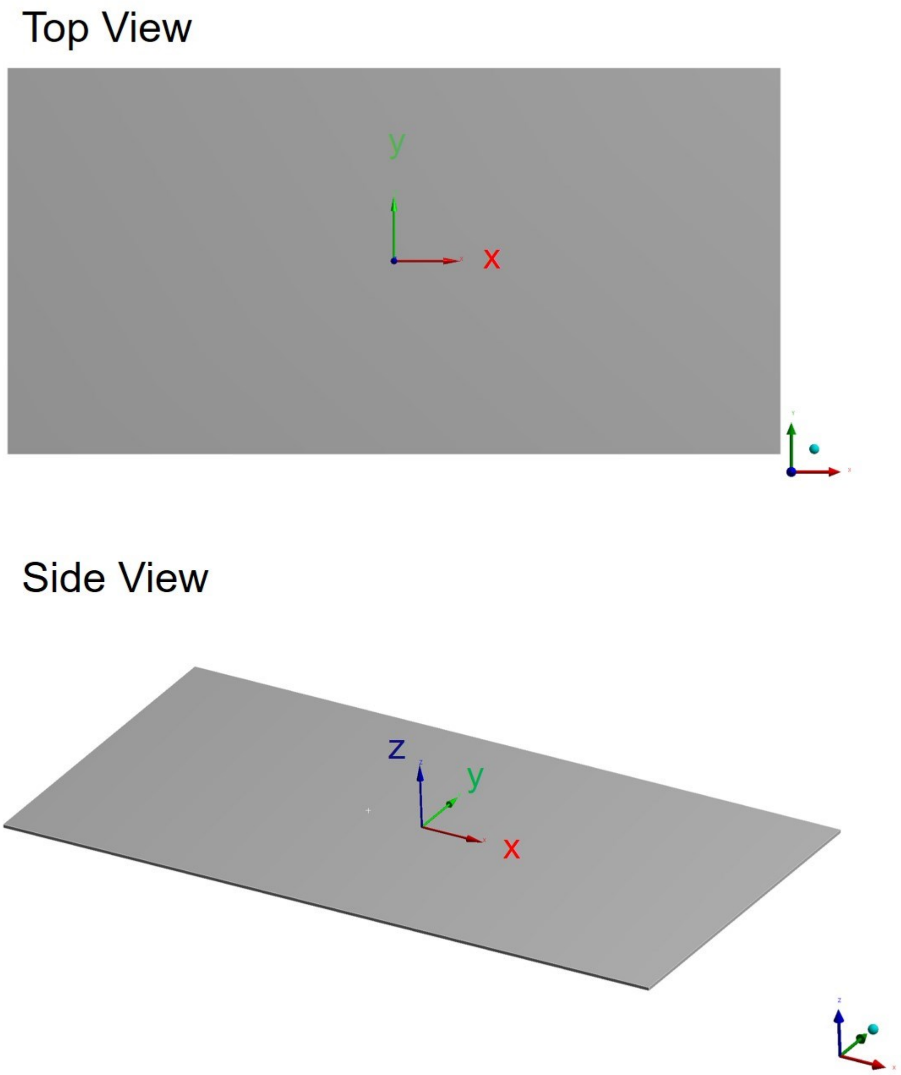

Supplementary Fig. 40 Cartesian coordinates and boundary conditions of FEA simulation. During the FEA simulation, the left boundary of FEA model was fixed, and the rest boundaries were set to be movable along *x* and *y* directions yet to with zero displacement along *z*-direction (the thickness direction).

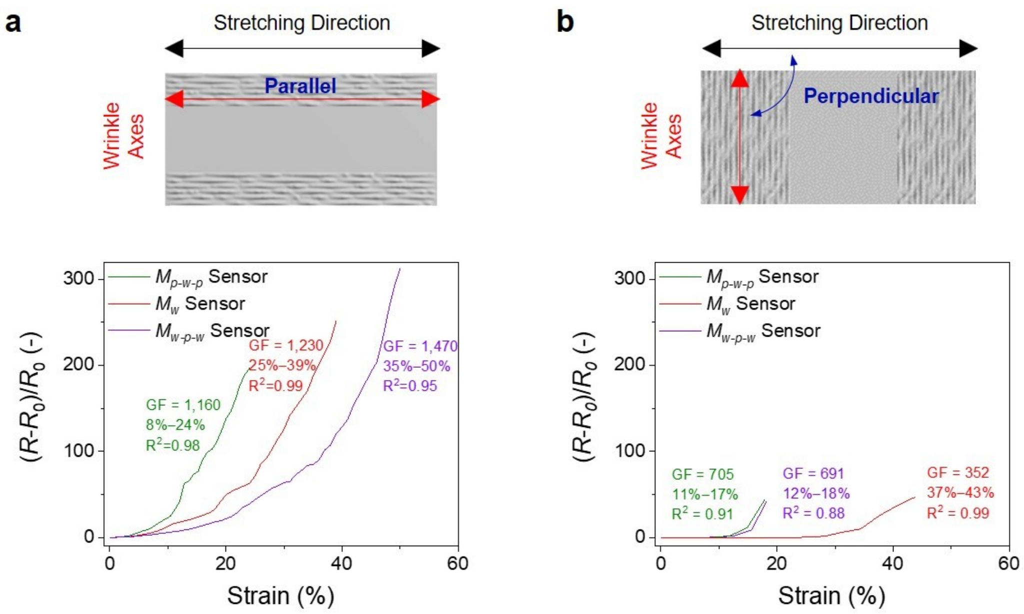

Supplementary Fig. 41 M_n sensors' performance under different stretching directions. (**a**) The stretching direction is parallel to the wrinkle axes. (**b**) The stretching direction is perpendicular to the wrinkle axes.

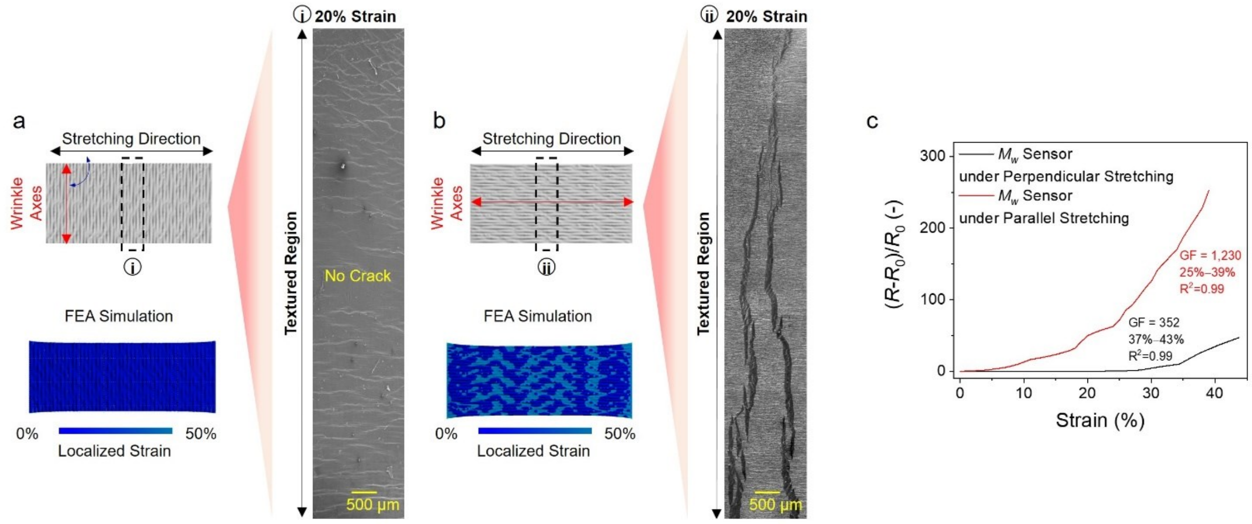

Supplementary Fig. 42 M_w sensor performance under different stretching directions. (**a**) FEA simulation and *in situ* SEM image of M_w nanolayer under perpendicular stretching. (**b**) FEA simulation and *in situ* SEM image of M_w nanolayer under parallel stretching. (**c**) Strain sensing curves of M_w sensor under perpendicular and parallel stretching.

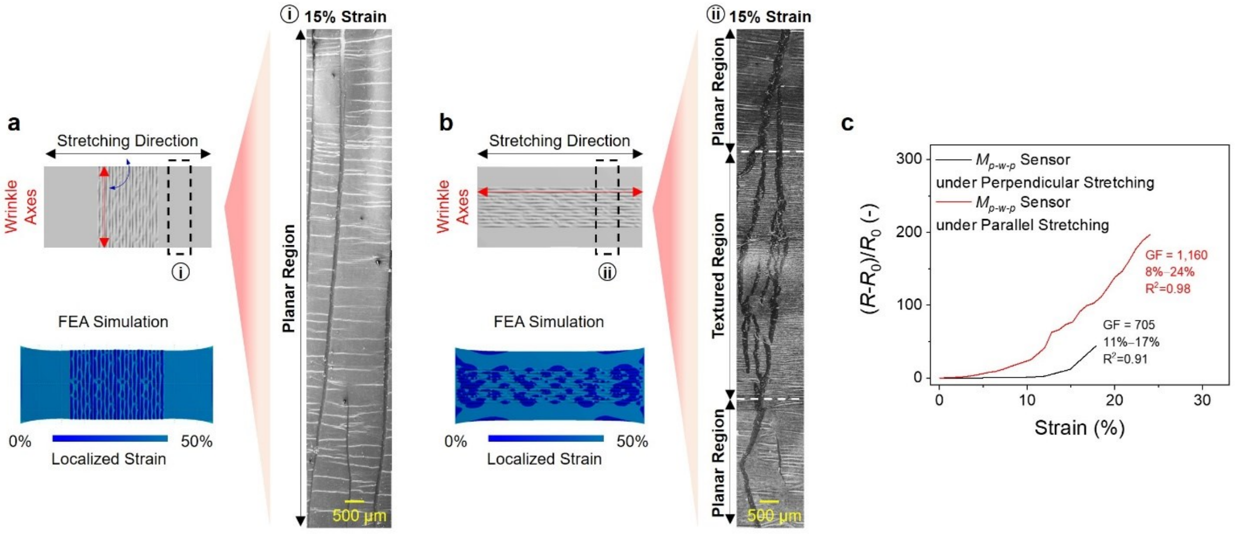

Supplementary Fig. 43 $M_{p\text{-}w\text{-}p}$ sensor performance under different stretching directions. (**a**) FEA simulation and *in situ* SEM image of $M_{p\text{-}w\text{-}p}$ nanolayer under perpendicular stretching. (**b**) FEA simulation and *in situ* SEM image of $M_{p\text{-}w\text{-}p}$ nanolayer under parallel stretching. (**c**) Strain sensing curves of $M_{p\text{-}w\text{-}p}$ sensor under perpendicular and parallel stretching.

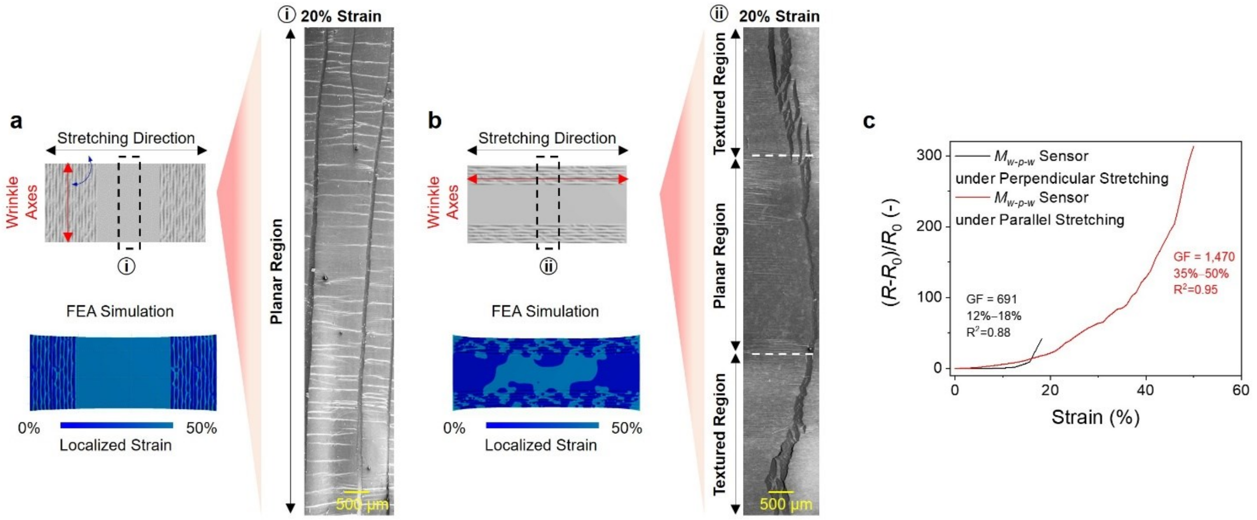

Supplementary Fig. 44 $M_{w\text{-}p\text{-}w}$ sensor performance under different stretching directions. (**a**) FEA simulation and *in situ* SEM image of $M_{w\text{-}p\text{-}w}$ nanolayer under perpendicular stretching. (**b**) FEA simulation and *in situ* SEM image of $M_{w\text{-}p\text{-}w}$ nanolayer under parallel stretching. (**c**) Strain sensing curves of $M_{w\text{-}p\text{-}w}$ sensor under perpendicular and parallel stretching.

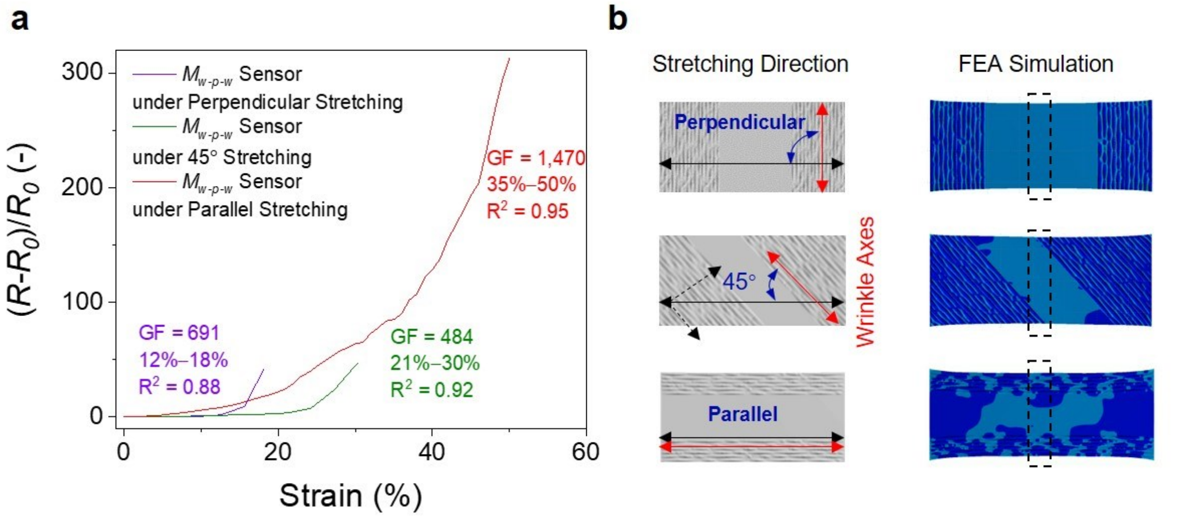

Supplementary Fig. 45 *$M_{w\text{-}p\text{-}w}$* sensor performance under different stretching directions. (**a**) Strain sensing curves of $M_{w\text{-}p\text{-}w}$ sensor under perpendicular, 45°, and parallel stretching. (**b**) FEA simulation of $M_{w\text{-}p\text{-}w}$ nanolayer under perpendicular, 45°, and parallel stretching.

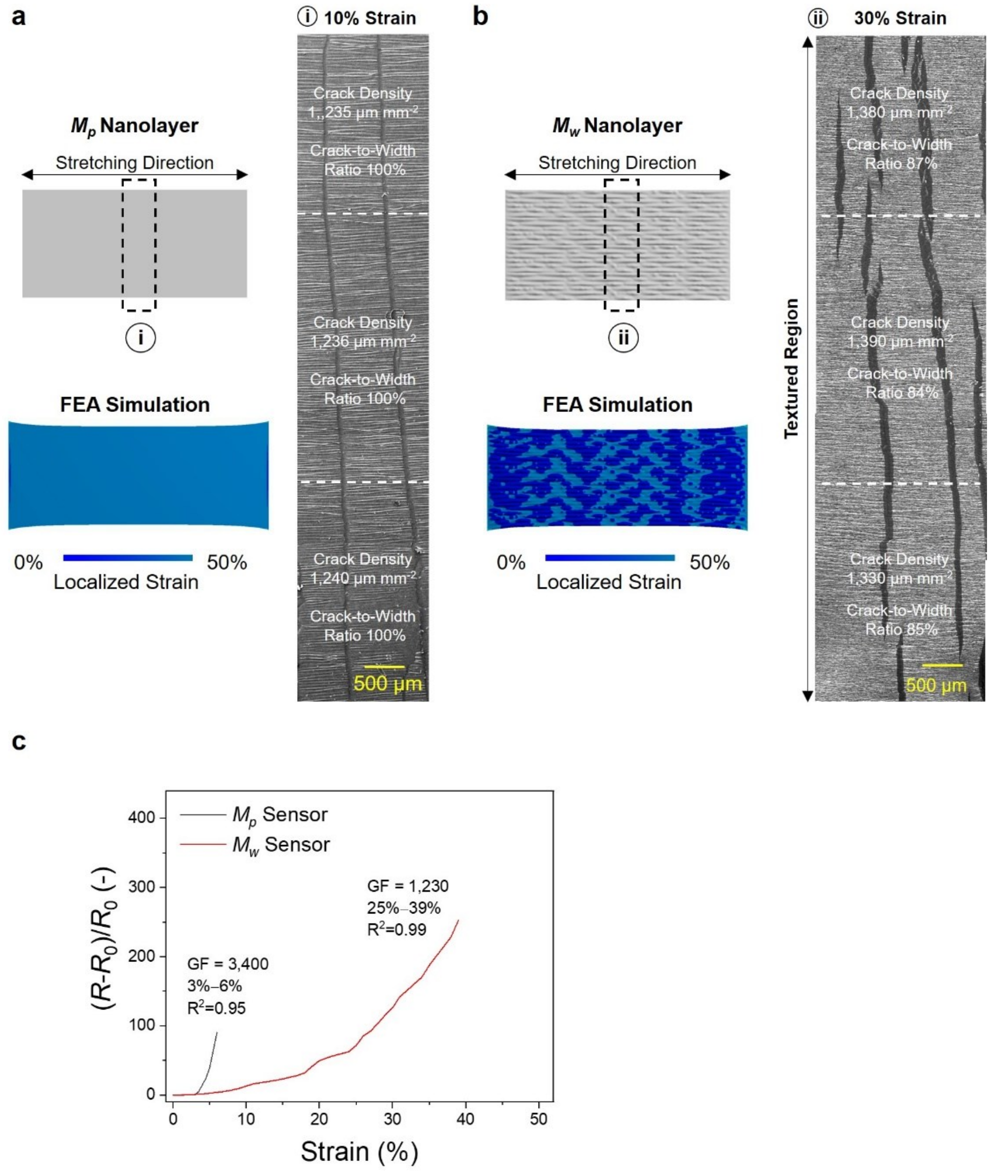

Supplementary Fig. 46 Performance comparison between M_p and M_w sensors. (**a**) FEA simulation and *in situ* SEM image of a M_p nanolayer under parallel stretching. (**b**) FEA simulation and *in situ* SEM image of a M_w nanolayer under parallel stretching. (**c**) Strain sensing curves of M_p and M_w sensors.

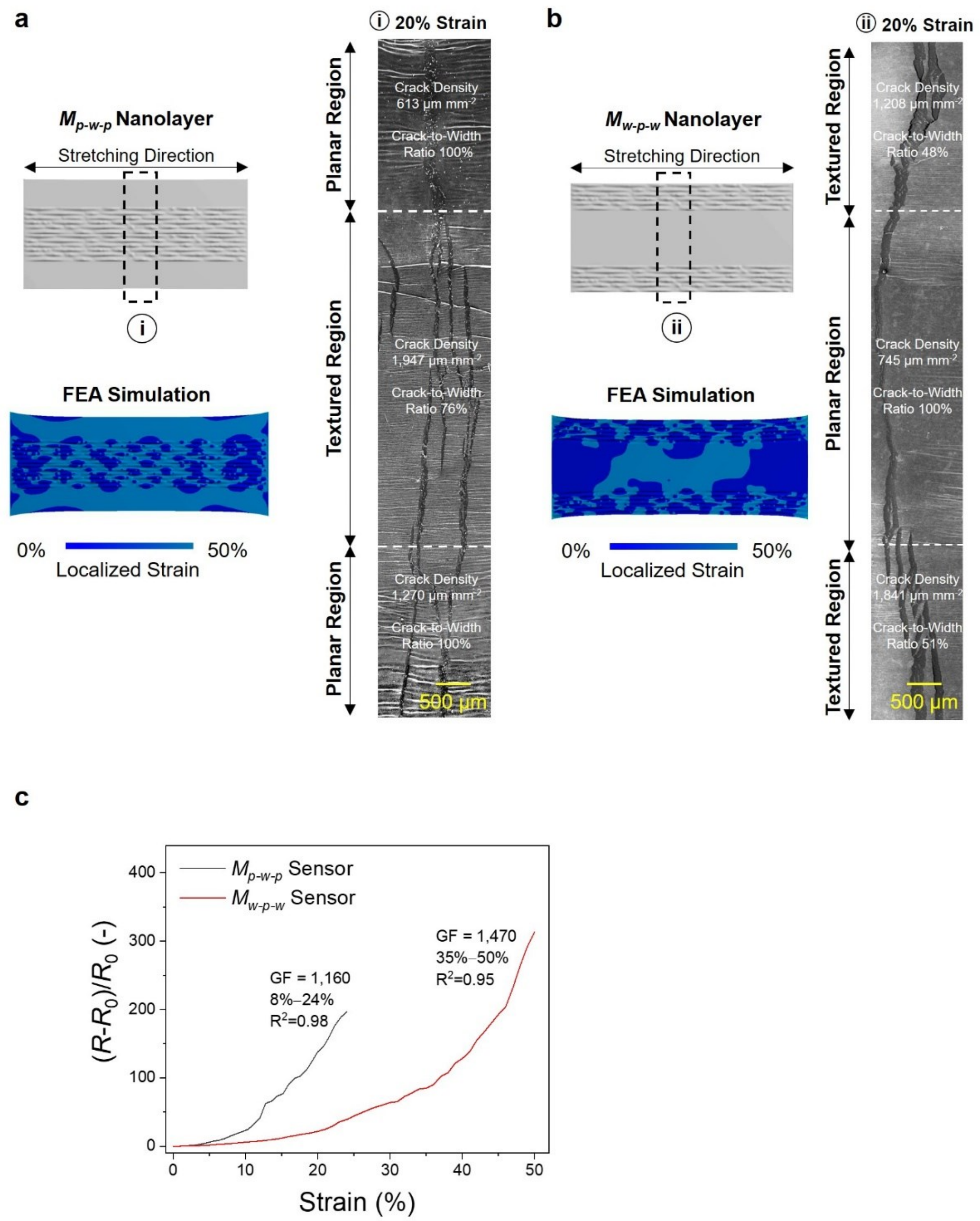

Supplementary Fig. 47 Performance comparison between $M_{p\text{-}w\text{-}p}$ and $M_{w\text{-}p\text{-}w}$ sensors. (**a**) FEA simulation and *in situ* SEM image of a $M_{p\text{-}w\text{-}p}$ nanolayer under parallel stretching. (**b**) FEA simulation and *in situ* SEM image of a $M_{w\text{-}p\text{-}w}$ nanolayer under parallel stretching. (**c**) Strain sensing curves of $M_{p\text{-}w\text{-}p}$ and $M_{w\text{-}p\text{-}w}$ sensors.

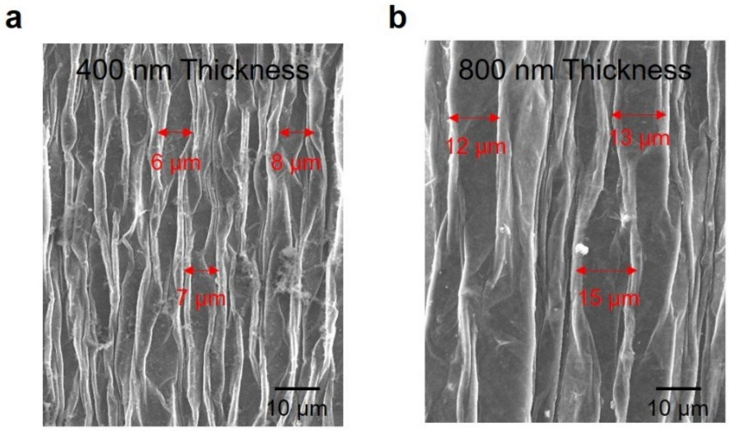

Supplementary Fig. 48 By increasing the nanolayer thickness from 400 to 800 nm, the wrinkle wavelength of M_w nanolayer increased from ca. 7 to 13 µm. The M_w nanolayers kept the same MXene/SWNT/PVA ratio of 65/30/5.

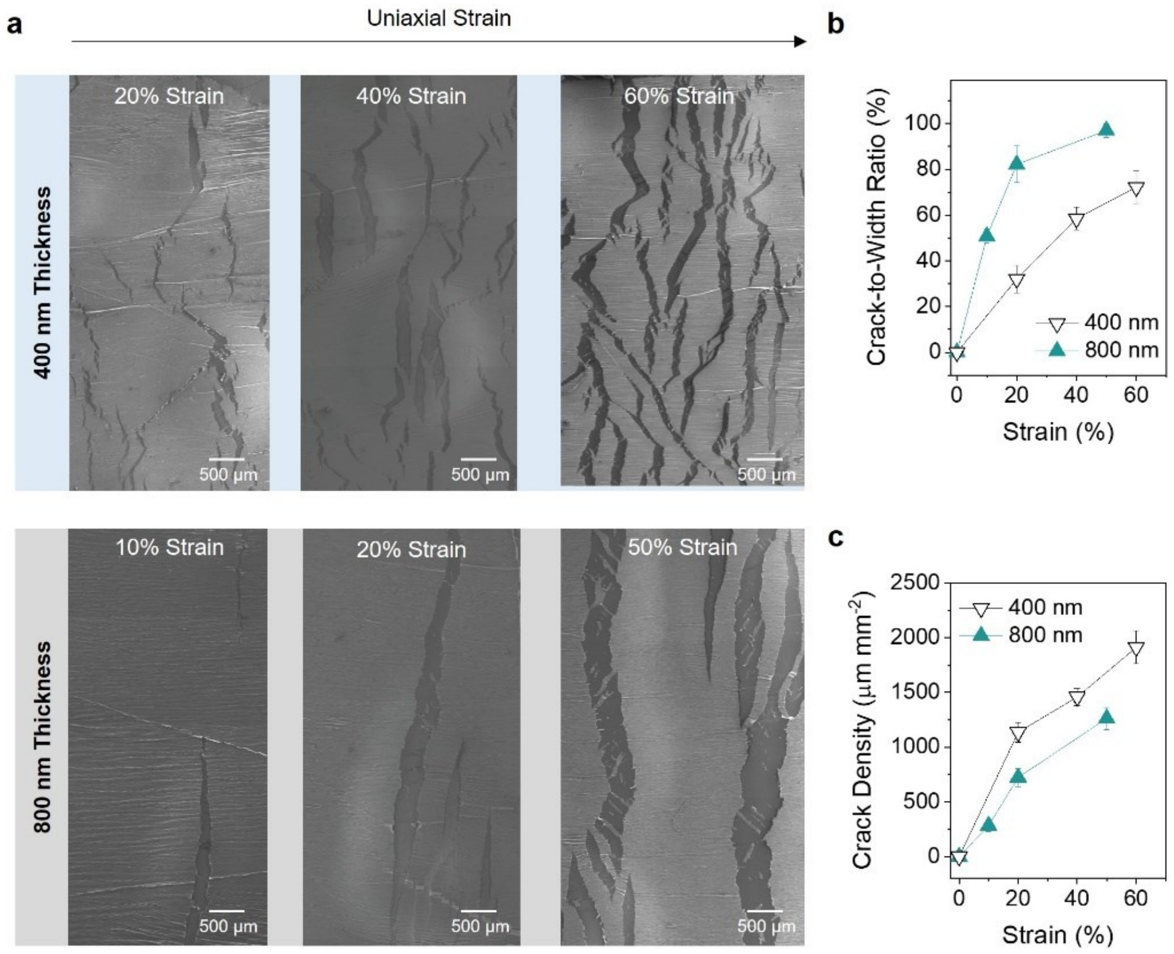

Supplementary Fig. 49 Crack propagation of M_w nanolayers with varying thicknesses under various strains. (**a**) SEM images of M_w nanolayers with varying thicknesses under various strains. (**b**) Crack-to-width ratios and (**c**) crack densities of M_w nanolayers with different thicknesses. The M_w nanolayers kept the same MXene/SWNT/PVA ratio of 65/30/5.

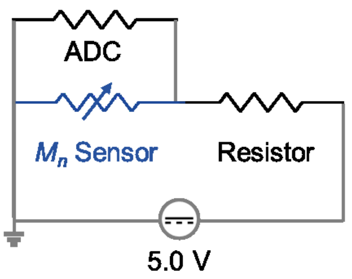

Supplementary Fig. 50 Equivalent circuit of the connections among a M_n sensor, ADC, and standard resistor.

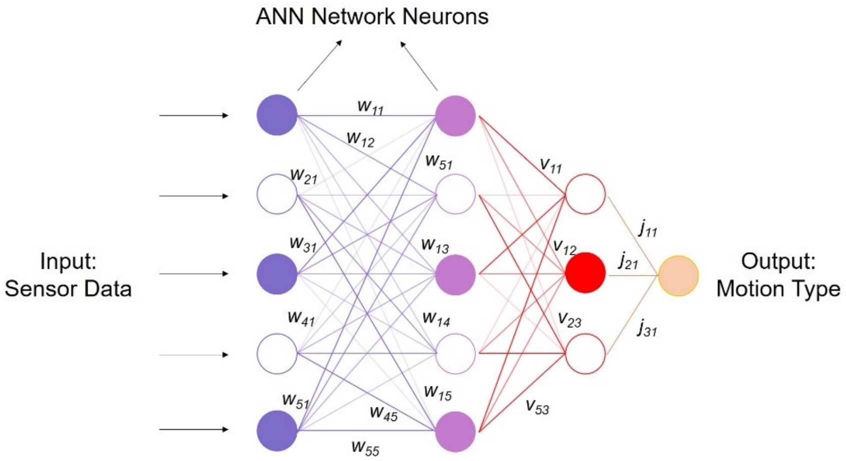

Supplementary Fig. 51 Working mechanism of ANN model.

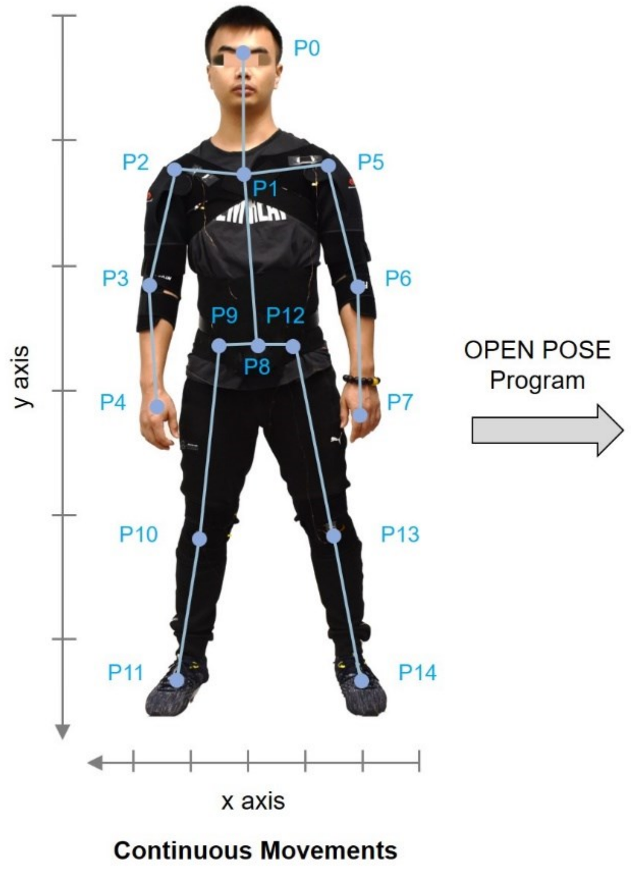

Time (s)	0	0.04	0.08	0.12	...
**P0 x**	792.2	792.2	792.2	792.2	...
**P0 y**	106.4	106.6	109.4	109.4	...
**P1 x**	809.9	809.9	809.9	809.9	...
**P1 y**	265.4	265.4	268.2	265.4	...
**P2 x**	709.8	709.8	709.8	709.8	...
**P2 y**	271.2	271.2	271.3	271.2	...
**P3 x**	692.1	692.1	692.1	692.1	...
**P3 y**	427.2	430.1	421.4	427.2	...
**P4 x**	671.7	671.7	671.7	671.7	...
**P4 y**	571.4	568.5	565.6	565.6	...
**P5 x**	907.1	907.1	907.1	907.1	...
**P5 y**	253.6	253.6	256.6	250.7	...
**P6 x**	954.2	954.2	954.2	954.2	...
**P6 y**	409.6	409.6	409.7	409.6	...
**P7 x**	968.8	968.8	968.8	968.8	...
**P7 y**	553.8	553.7	551.0	550.9	...
**P8 x**	824.6	824.6	824.6	824.6	...
**P8 y**	545.0	547.9	550.8	548.0	...
**P9 x**	762.7	762.7	762.7	762.7	...
**P9 y**	545.0	548.0	550.8	548.0	...
**P10 x**	700.0	700.0	700.0	700.0	...
**P10 y**	745.2	745.1	745.2	745.1	...
**P11 x**	742.1	742.1	742.1	742.1	...
**P11 y**	954.0	951.2	951.3	951.3	...
**P12 x**	886.4	886.4	886.4	886.4	...
**P12 y**	545.0	548.0	550.8	548.0	...
**P13 x**	970.6	970.6	970.6	970.6	...
**P13 y**	745.1	742.3	742.3	742.2	...
**P14 x**	951.2	951.2	951.2	951.2	...
**P14 y**	945.2	936.5	939.4	936.4	...

Supplementary Fig. 52 Working mechanism of OPEN POSE program.

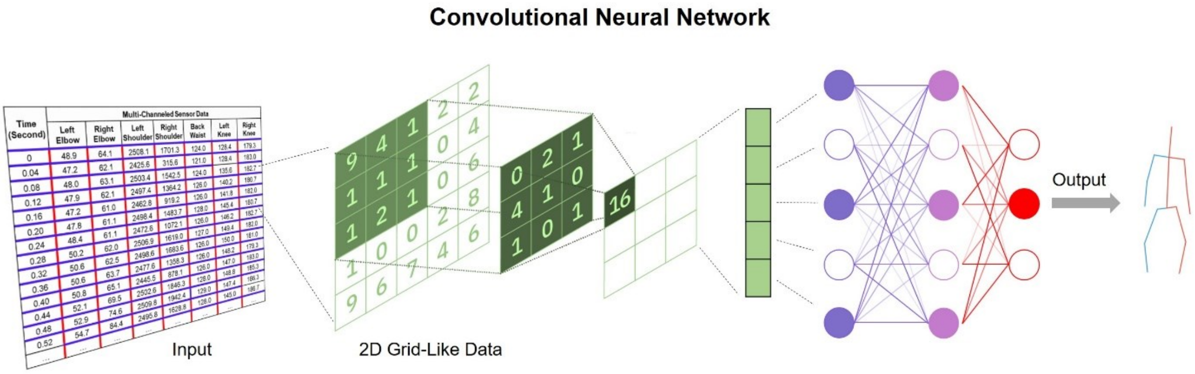

Supplementary Fig. 53 Working mechanism of CNN model.

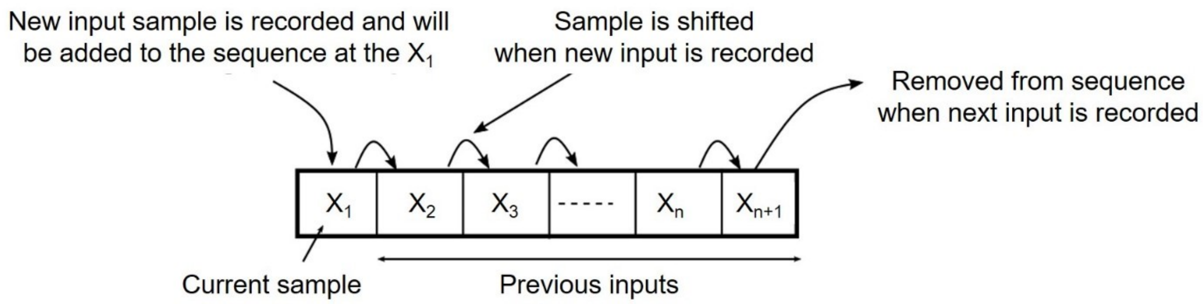

Fig.54 Implementation of FIFO register for real-time computation using a CNN model.

a

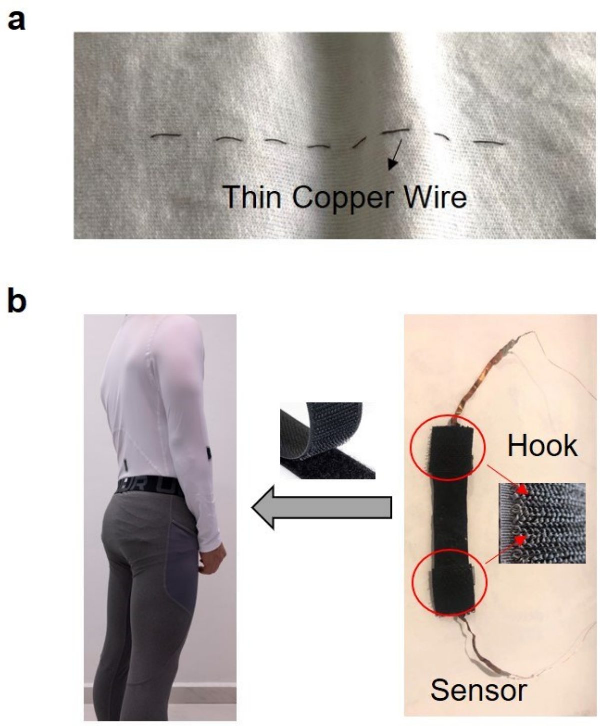

Supplementary Fig. 55 Increasing comfort level of wearing sensor modules. (**a**) Copper wires were sewed into the fabrics to avoid circuit disorders. (**b**) Commercial hook-and-loop fasteners were used to stabilize the M_n sensors on the clothing.

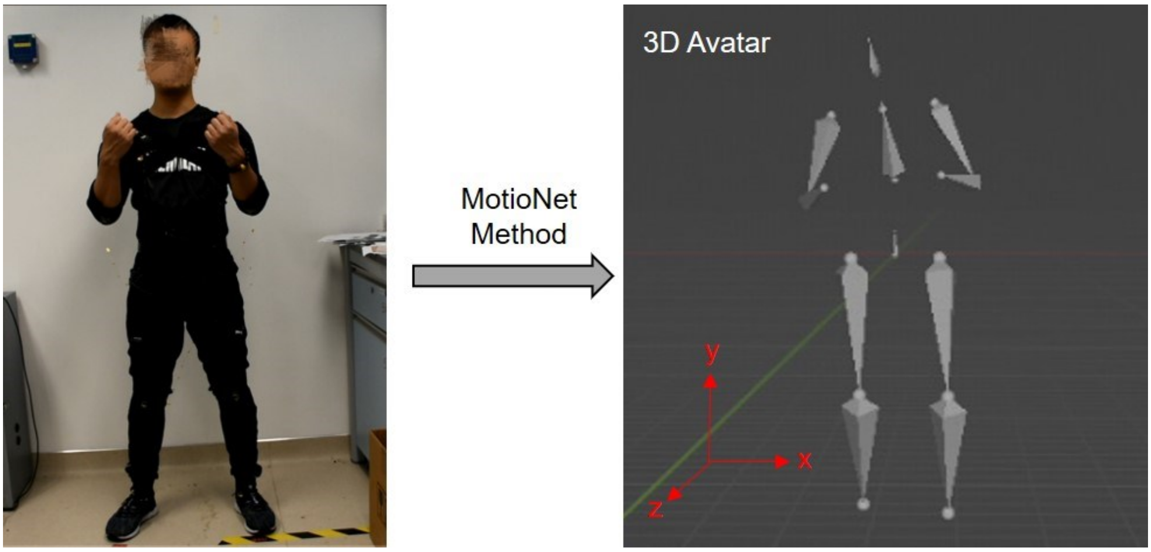

Supplementary Fig. 56 Reconstruction of a 3D avatar *via* MotioNet method.

Supplementary Note **1. The effects of stretching directions on the crack propagation behaviors of M_n sensors.**

There are two major advantages of selecting parallel stretching over the perpendicular stretching, including **(1)** higher M_n sensors' sensitivities and wider linear working windows and **(2)** more design opportunities *via* topographic design. As shown in **Supplementary Fig. 41**, the M_n sensors (including M_w, $M_{w\text{-}p\text{-}w}$, $M_{p\text{-}w\text{-}p}$) under parallel stretching demonstrated wider linear working windows (with an average strain range of 15%) and higher sensitivities (GF >1,100) than the M_n sensors under perpendicular stretching (an average strain range of 6%, GF ~600).

To investigate the effect of stretching directions (perpendicular, 45°, and parallel) on the crack propagation behaviors of all M_n sensors (from M_w to $M_{p\text{-}w\text{-}p}$ and $M_{w\text{-}p\text{-}w}$), additional FEA simulation and *in situ* SEM have been conducted in the revised manuscript.

First, for the M_w sensor, **Supplementary Fig. 42a** and **42b** show the FEA and *in situ* SEM results under perpendicular and parallel stretching. When stretched perpendicularly, the M_w nanolayer experienced attenuated strains (<10%, quantified by *ImageJ*), and the periodic wrinkles were first unfolded into a planar morphology and then quickly formed large, long cracks (**Supplementary Fig. 42a**). Thus, the M_w sensor under perpendicular stretching demonstrated a relatively lower sensitivity (GF <400) and a narrower linear working window of 37–43% (**Supplementary Fig. 42c**). In comparison, when stretched parallelly, the M_w nanolayer experienced moderate strains (ca. 20%), and short/zigzag cracks propagated accordingly (**Supplementary Fig. 42b**). The M_w sensor under perpendicular stretching demonstrated a higher GF of 1,230 and a wider linear working window of 25%–39% (**Supplementary Fig. 42c**).

Second, for the $M_{p\text{-}w\text{-}p}$ sensor, **Supplementary Fig. 43a** and **43b** show the FEA and *in situ* SEM results under perpendicular and parallel stretching. When the $M_{p\text{-}w\text{-}p}$ nanolayer was

stretched perpendicularly, the side planar regions experienced large strains (>50%), and long cracks emerged and quickly cut off the conductive pathways (**Supplementary Fig. 43a**). As a result, the $M_{p\text{-}w\text{-}p}$ sensor under perpendicular stretching demonstrated a relatively lower sensitivity (GF <700) and a narrower linear working window of 11–17% (**Supplementary Fig. 43c**). In comparison, when the $M_{p\text{-}w\text{-}p}$ nanolayer was stretched parallelly, the middle region (with wrinkle-like textures) experienced attenuated strains (<20%) and developed short/zigzag cracks. Although the side planar regions showed long and continuous cracks, the middle region prevented the conductive pathways from being completely cut off (**Supplementary Fig. 43b**). As a result, the $M_{p\text{-}w\text{-}p}$ sensor under perpendicular stretching showed a higher GF of 1,160 and a wider linear working window of 8–24% (**Supplementary Fig. 43c**).

Third, for the $M_{w\text{-}p\text{-}w}$ sensor, **Supplementary Fig. 44a** and **44b** show the FEA and *in situ* SEM results under perpendicular and parallel stretching. The $M_{w\text{-}p\text{-}w}$ sensor demonstrated similar crack propagation behaviors as the $M_{p\text{-}w\text{-}p}$ sensor. When the $M_{w\text{-}p\text{-}w}$ nanolayer was stretched perpendicularly, the middle planar region developed long cracks that quickly cut off the conductive pathways (**Supplementary Fig. 44a**). As a result, the $M_{w\text{-}p\text{-}w}$ sensor under perpendicular stretching demonstrated a narrower linear working window of 12–18% (**Supplementary Fig. 44c**). In comparison, when stretched parallelly, the $M_{w\text{-}p\text{-}w}$ nanolayer experienced attenuated strains (<20%) in the edge regions (with wrinkle-like textures), where short/zigzag cracks emerged. Although the middle planar region showed long and continuous cracks, the edge regions prevented the conductive pathways from being completely cut off (**Supplementary Fig. 44b**). As a result, the linear working window of a $M_{w\text{-}p\text{-}w}$ sensor expanded to 35–50% by changing the stretching direction (**Supplementary Fig. 44c**).

Furthermore, for the $M_{w\text{-}p\text{-}w}$ sensor, additional studies were carried out under 45° stretching in **Supplementary Fig. 45**. When the $M_{w\text{-}p\text{-}w}$ nanolayer was under 45° stretching, the strain was divided into two directions: one is parallel, and the other is perpendicular to the

wrinkle axes. The $M_{w\text{-}p\text{-}w}$ nanolayer under 45° stretching demonstrated the FEA results with mixed effects. According to the results from **Supplementary Fig. 42** to **44**, the M_n sensors under parallel stretching normally demonstrated wider working windows, and the M_n sensors under perpendicular stretching showed lower sensitivities (i.e., GF). As a result, as shown in **Supplementary Fig. 45**, the $M_{w\text{-}p\text{-}w}$ sensor under 45° stretching showed a moderate working window of 21–30% yet a low GF of 484.

Considering the advantages of higher strain sensitivities and wider linear working windows, we adopted the parallel stretching direction for all M_n sensors.

Supplementary Note 2. Comparison of M_n sensors' performance.

There are two categories of M_n nanolayers developed in this work: (1) the ps-MXene nanolayers with homogenous topographies (including M_p and M_w) and (2) the ps-MXene nanolayers with heterogeneous topographies (including $M_{p\text{-}w\text{-}p}$ and $M_{w\text{-}p\text{-}w}$).

According to the FEA results in **Supplementary Fig. 46**, the M_p and M_w nanolayers exhibited homogenous localized strain distribution profiles. Under parallel stretching, the M_p and M_w nanolayers exhibited similar in-plane crack densities and crack-to-width ratios (see definitions in **Methods**) between the edge and middle regions. On the other hand, from the FEA results in **Supplementary Fig. 47**, the $M_{p\text{-}w\text{-}p}$ and $M_{w\text{-}p\text{-}w}$ nanolayers showed region-dependent and heterogenous strain distribution profiles. Under parallel stretching, the $M_{p\text{-}w\text{-}p}$ and $M_{w\text{-}p\text{-}w}$ nanolayers showed distinct in-plane crack densities and crack-to-width ratios between the edge and middle regions. Because the FEA result could not reflect the regional mismatch of localized strains and the complexity of transition regions (between planar and wrinkle-like regions, **Supplementary Fig. 8**), we only compared the M_n sensors in the same

category. The comparison (1) between M_p and M_w sensors (homogenous topographies) and (2) between $M_{p\text{-}w\text{-}p}$ and $M_{w\text{-}p\text{-}w}$ sensors (heterogenous topographies) are provided as follows.

First, the M_p and M_w sensors with homogenous topographies were compared. Based on the FEA results in **Supplementary Fig. 46**, the M_p nanolayer under 120% stretching showed an average localized strain of 49% (quantified by *ImageJ*), while the M_w nanolayer demonstrated a much lower average localized strain of 20%. As a result, the M_p sensor demonstrated a much smaller ε_{max} of 6% than the M_w sensor (ε_{max} = 39%).

Second, the $M_{p\text{-}w\text{-}p}$ and $M_{w\text{-}p\text{-}w}$ sensors with heterogenous topographies were compared. Based on the FEA results in **Supplementary Fig. 47**, the $M_{p\text{-}w\text{-}p}$ nanolayer under 120% stretching showed an average localized strain of 32% (quantified by *ImageJ*), while the $M_{w\text{-}p\text{-}w}$ nanolayer demonstrated a lower average localized strain of 20%. As a result, the $M_{w\text{-}p\text{-}w}$ sensor demonstrated a much smaller ε_{max} of 24% than the $M_{w\text{-}p\text{-}w}$ sensor (ε_{max} = 50%).

Supplementary Note 3. The effects of nanolayer thicknesses on the M_n sensors' morphologies and crack propagation behaviors.

Herein, *in situ* electron microscopic studies have been conducted to investigate the effect of nanolayer thicknesses on the M_n sensors' crack propagation behaviors and strain sensing performance. Taking the M_w sensor as an example in **Supplementary Fig. 48**, by increasing the nanolayer thicknesses from 400 to 800 nm, the average wrinkle wavelength of M_w nanolayer increased from ca. 7 to 13 μm. With different wrinkle wavelengths, the M_w sensors exhibited different crack propagation behaviors. **Supplementary Fig. 49a** recorded the *in situ* SEM images of two M_w sensors (with 800- and 400-nm-thick nanolayers) under parallel stretching. As summarized in **Supplementary Fig. 49b**, the crack-to-width ratio (see definition in **Methods**) of the M_w sensor with a thicker nanolayer increased faster than the one with a thinner nanolayer. In addition, in **Supplementary Fig. 49c**, the maximal crack density (see

definition in **Methods**) of the M_w sensor with a thinner nanolayer was 1,920 μm mm^{-2}, larger than the one with a thicker nanolayer (1,260 μm mm^{-2}).

Supplementary Note 4. Transmission error of wireless sensor module.

As shown in **Supplementary Fig. 50**, the voltage across a M_n sensor was measured by an ADC unit. As the applied strains increased, the resistance of a M_n sensor increased accordingly from 0.1 to 100 kΩ, and the input impedance of an ADC unit was about 1 MΩ (see ADC data sheet in **GitHub**: https://github.com/Haitao008/Supporting-Tables). As a result, when the resistances of M_n sensors increased under strains, the total resistance that consisted of a parallel impedance combination of an ADC unit and M_n sensors changed from 0.09 to 90.9 kΩ, leading to the resistance deviations of M_n sensors from 0.1 to 9.0%. It is worth noting that, as shown in **Eqns. S1** and **12**, the input impedance of the ADC unit had been considered and included when calculating the output resistance values of a M_n sensor from the ADC-recorded voltage values as well as estimating the transmission errors. In other words, when calculating the transmission error, we had already considered the resistance deviations induced by the ADC unit.

$$R_{output} = \frac{V_{ADC} \times R_{standard} \times R_{ADC}}{5 \times R_{ADC} - V_{ADC} \times (R_{standard} + R_{ADC})} \quad \textbf{(S1)}$$

, where R_{output} is the estimated resistance of a M_n sensor, V_{ADC} is ADC-measured voltage across a M_n sensor, 5 is the applied voltage, R_{ADC} is the input impedance of an ADC (i.e., 1 MΩ), and $R_{standard}$ is the resistance of an integrated standard resistor.

Under an applied voltage of 5.0 V, the voltage read from ADC across a M_n sensor is dependent on the value of the standard resistor connected in series (see the circuit in **Fig. 4g**). **Supplementary Table 7** compares the voltages read from ADC across a M_n sensor (with strain-dependent resistances), which was connected in series with a 5- or 100-kΩ standard resistor. As shown in **Supplementary Table 7**, if a 100-kΩ standard resistor was connected, the voltages read from the ADC across a M_n sensor increased from 1.61 to 2.26 V, when the M_n sensor's resistance increased with strains from 50 to 90 kΩ. On the other hand, if a 5-kΩ standard resistor was connected, the voltage read from ADC across a M_n sensor only increased

from 4.52 to 4.71 V, when the M_n sensor's resistance increased with strains from 50 to 90 kΩ. The small voltage changes were affected severely by the noise signals and caused the fluctuations of ADC-measured voltages, leading to the wrong estimations of M_n sensor resistance values. The transmission error was mainly because of the noise associated with reading voltage with discrete ADC and wireless modules.

It is worthy to mention that, by integrating tunable impedance into the circuit, we could avoid the selection of optimal standard resistors to accompany M_n sensors and further achieve tight circuit integration (with M_n sensors, ADC, and wireless/edge computing modules), which can reduce the noise associated with discrete devices.

Supplementary Note 5. Artificial Neural Network (ANN) model.

As shown in **Supplementary Fig. 51**, the ANN model is constructed by a bunch of neuron base units with learning parameters (e.g., w_{12}, w_{15}). Similar as other supervised machine learning (ML) algorithms, the ANN model aims to use a set of training data to predict an output based on the inputs.[1] In this study, the inputs are the multi-channeled M_n sensor data, and the output is a classified full-body motion. The training data are the multi-channeled M_n sensor data with corresponding full-body motions. The multi-channeled M_n sensor data were collected by the wireless sensor module, and the vectors with six dimensions were used to encode six different full-body motions (e.g., 000001 for motion i, 000010 for motion ii, 000100 for motion iii). An activation function, Softmax, was implemented at the output layer, and the cross-entropy loss was utilized as the loss function. 10-fold cross-validation was implemented for the training of an ANN model. Afterward, a set of test data were used to evaluate the prediction accuracy of the trained ANN model, which was confirmed to be 100%.

The ANN model training was implemented by using Keras framework in Python, which can be found in **GitHub** (https://github.com/jiali1025/Wearable-MXene-Sensors-with-In-Sensor-Machine-Learning-for-Full-Body-Avatar-Reconstruction).

Supplementary Note 6. t-distributed Stochastic Neighbor Embedding (t-SNE).

In order to visualize and cluster the multi-channeled M_n sensor data from various full-body motions, t-SNE,[2] a renowned nonlinear multivariate data dimension reduction and visualization method,[3-5] was conducted. By extracting the comprehensive characteristics of these M_n sensor signals, t-SNE produces significantly better visualizations by reflecting the sensor data similarity with clusters of points.

In principle, t-SNE converts the affinities of data points in original space into Gaussian joint probabilities in lower dimension. However, there always exists divergence between the probability distributions P (i.e., true distribution of the data) and Q (i.e., theoretical distribution or approximation distribution), which could be reflected by the Kullback-Leibler (KL) divergence. The KL divergence measures the asymmetry of the difference between P and Q, and also the additional average number of bits required to use a Q-based distribution to encode samples that obey P distribution.[6] As such, we minimized the KL divergence by gradient descent to ensure that the low-dimensional probabilities truly reflected the high-dimensional affinities of data points. After the information of complex patterns was reduced in dimensionality, a more intuitive plot comparing the various patterns was obtained, which was the low-dimensional mapping of high-dimensional space (i.e., t-SNE dimensions 1 and 2 in this work).

The t-SNE dimension reduction was implemented by using Sklearn framework in Python, which can be found in **GitHub** (https://github.com/jiali1025/Wearable-MXene-Sensors-with-In-Sensor-Machine-Learning-for-Full-Body-Avatar-Reconstruction).

Supplementary Note 7. OPEN POSE program.

OPEN POSE is an open-source program to extract the joint locations of a volunteer from an image/video.[7] As shown in **Supplementary Fig. 52**, OPEN POSE extracted 15 important joint locations (from P0 to P14) from the image/video of a volunteer, and a stationary stickman avatar was constructed. Each joint had a defined position to reflect its spatial location, and the full-body motion video clip was converted into a text file with the x–y joint locations. The joint locations were coupled with the corresponding M_n sensor data as the training data for a prediction model. Afterward, a set of test data (M_n sensor data and x–y joint locations) were used to evaluate the model's prediction accuracy.

Supplementary Note 8. Convolutional Neural Network (CNN) model.

CNN is a specialized form of neural networks, which is specifically powerful in processing the grid-like data (such as audio waveforms and 2D/3D images).[8] As shown in **Supplementary Fig. 53**, the multi-channeled M_n sensor data with a fixed time length were categized as the grid-like data and processed by CNN. It is worth to note that, in this work, the CNN kernel size was selected using Bayesian optimization (see reference in **GitHub**: https://github.com/fmfn/BayesianOptimization). Bayesian optimization enables hyperparameter tuning without the need for grid search. Additionally, for the prediction of full-body motion movements, the Bayesian optimizer tunes the time length of newly input sensor data by considering the history of previous sensor data. As shown in **Supplementary Fig. 54**,

the First-in-First-out (FIFO) register stores the previously input sensor data and bundle them with the new input data, and the data points (past + current) are used to predict the avatar movement. In this work, the end-to-end regression with CNN took less than 1 millisecond.

In this study, the training data composed of M_n sensor data and x–y joint locations were utilized to construct a CNN model. After the model training was completed, the CNN model was programmed into an ARDUINO chip (Element14 Pte Ltd) to perform the edge computing to realize in-sensor full-body avatar reconstruction in real time. The CNN model training was implemented by using Keras framework in Python, which can be found in **GitHub** (https://github.com/jiali1025/Wearable-MXene-Sensors-with-In-Sensor-Machine-Learning-for-Full-Body-Avatar-Reconstruction).

Supplementary Note 9 Discussion of avatar animation moving ahead of squatting motions.

As shown in **Supplementary Fig. 36** and **Supplementary Movie 4**, the avatar's squatting movement (at the 22.6th second) was ahead of the video-recorded squatting motion (at the 23.0th second). The ahead motion determination was due to the early signals from the M_p sensor on the back waist (at the 22.4th second). In **Supplementary Fig. 36**, before the squatting motion, the M_p sensor on the back waist (at the P8 joint) was able to sense the preparatory muscular stretching (at the 22.4th second), and the M_p sensor data were recognized as the early signals for a squatting motion. The early M_p sensor signals were observed every time during the repeated squatting motions, and the M_p sensor reached the peaks before the squatting motions were finished. Therefore, the CNN model would determine the avatar's squatting motions ahead of time (ca. 0.4 second). The ahead avatar animation was a clear evidence that the M_n sensors with high sensitivities and customized working windows are suitable for detecting delicate muscle movements.

Supplementary Note 10 Improving comfort levels of wearing M_n sensor modules.

To increase the comfort levels of wearing the M_n sensor modules, two approaches have been developed in the revised manuscript. First, as shown in **Supplementary Fig. 55a**, the copper wires were sewed into the fabrics to avoid circuit disorders over the human body and limbs. Second, the M_n sensors were further stabilized on the clothing using commercial hook-and-loop fasteners, which provided sufficient mechanical stability and became convenient to be attached/detached or adjusted (**Supplementary Fig. 55b**).

Supplementary Note 11 Construction of 3D avatar animation.

As shown in **Supplementary Fig. 56** and **Supplementary Movie 5**, by adopting the "MotioNet" method from the computer vision field (see more details in Reference[9]), a 3D avatar was constructed based on the 2D avatar results determined by the edge sensor module. The working mechanism of MotioNet is similar to the OPEN POSE program. First, MotioNet extracted the full-body motions from the recorded videos and estimated 15 joint locations of a volunteer in *x*, *y*, and *z* coordinates. As shown in **Supplementary Fig. 56**, a stationary 3D avatar with 15 joints was then constructed. Afterward, based on the M_n sensor database, the CNN model was re-trained to output the joint locations in *x*, *y*, and *z* coordinates, which were utilized by MotioNet to re-construct the 3D avatar's animation.

Supplementary Table 1. Fabrication parameters of 12 M_n sensors in Fig. 3i, including nanolayer morphologies, compositions, and thicknesses.

Index	Nanolayer Morphology (–)	MXene/SWNT/PVA Ratio (wt.%)	Nanolayer Thickness (nm)
I	M_p	85/10/5	400
II	$M_{p\text{-}w\text{-}p}$	85/10/5	400
III	M_w	85/10/5	400
IV	$M_{w\text{-}p\text{-}w}$	85/10/5	400
V	M_p	65/30/5	400
VI	$M_{p\text{-}w\text{-}p}$	65/30/5	400
VII	M_w	65/30/5	400
VIII	$M_{w\text{-}p\text{-}w}$	65/30/5	400
IX	M_p	65/30/5	800
X	$M_{p\text{-}w\text{-}p}$	65/30/5	800
XI	M_w	65/30/5	800
XII	$M_{w\text{-}p\text{-}w}$	65/30/5	800

Supplementary Table 7 Comparison of ADC-measured voltages using different standard resistor values of 5 kΩ and 100 kΩ.

Resistance of a M_n Sensor	Voltage read from ADC (calculated with 5-kΩ standard resistance)	Voltage read from ADC (calculated with 100-kΩ standard resistance)
50 kΩ	4.52 V	1.61 V
60 kΩ	4.60 V	1.81 V
70 kΩ	4.65 V	1.98 V
80 kΩ	4.68 V	2.13 V
90 kΩ	4.71 V	2.26 V

Supplementary References

1 Jain, A. K., Jianchang, M. & Mohiuddin, K. M. Artificial neural networks: A tutorial. *Computer* **29**, 31-44, (1996).

2 Maaten, L. v. d. & Hinton, G. Visualizing data using t-SNE. *J. Mach. Learn. Res.* **9**, 2579-2605, (2008).

3 Abdelmoula, W. M. *et al.* Data-driven identification of prognostic tumor subpopulations using spatially mapped t-SNE of mass spectrometry imaging data. *Proc. Natl. Acad. Sci.* **113**, 12244-12249, (2016).

4 Wong, M. T. *et al.* A high-dimensional atlas of human T cell diversity reveals tissue-specific trafficking and cytokine signatures. *Immunity* **45**, 442-456, (2016).

5 Kruiger, J. F. *et al.* Graph layouts by t-SNE. *Comput. Graph. Forum* **36**, 283-294, (2017).

6 Kullback, S. & Leibler, R. A. On information and sufficiency. *Ann. Math. Stat.* **22**, 79-86, (1951).

7 Cao, Z., Hidalgo, G., Simon, T., Wei, S.-E. & Sheikh, Y. Openpose: Realtime multi-person 2D pose estimation using part affinity fields. *IEEE PAMI* **43**, 172-186, (2019).

8 Jmour, N., Zayen, S. & Abdelkrim, A. in *2018 International Conference on Advanced Systems and Electric Technologies (IC_ASET).* 397-402 (IEEE).

9 Shi, M. *et al.* Motionet: 3D human motion reconstruction from monocular video with skeleton consistency. *ACM Trans. Graph.* **40**, 1-15, (2020).
