# Supplementary material for: Topographic design in wearable MXene sensors with in-sensor machine learning for full-body avatar reconstruction
Source: Nat Commun. 2022 Sep 9;13:5311. doi: 10.1038/s41467-022-33021-5 (PMC9461448; doi:10.1038/s41467-022-33021-5)
Supplement: Supplementary file 3 — Description of Additional Supplementary Files [file 41467_2022_33021_MOESM3_ESM.docx]

**Description of Additional Supplementary Files**

**File Name: Supplementary Movie 1
Description:** Crack propagation behaviors of Mp sensor under strain loading processes.

**File Name: Supplementary Movie 2
Description:** Crack propagation behaviors of Mw sensor under strain loading processes.

**File Name: Supplementary Movie 3
Description:** Crack propagation behaviors of Mw sensor under repeated uniaxial strain.

**File Name: Supplementary Movie 4
Description:** Comparison between edge-determined avatar animations and camera-recorded volunteer motions.

**File Name: Supplementary Movie 5
Description:** Comparison between edge-determined 3D avatar animations and camera-recorded volunteer motions.
